# Acknowledgment to the Reviewers of *Pharmaceutics* in 2022

**DOI:** 10.3390/pharmaceutics15020331

**Published:** 2023-01-19

**Authors:** Pharmaceutics Editorial Office

**Affiliations:** MDPI AG, St. Alban-Anlage 66, 4052 Basel, Switzerland

High-quality academic publishing is built on rigorous peer review. *Pharmaceutics* was able to uphold its high standards for published papers due to the outstanding efforts of our reviewers. Thanks to the efforts of our reviewers in 2022, the median time to first decision was 15.9 days and the median time to publication was 39 days. Regardless of whether the articles they examined were ultimately published, the editors would like to express their appreciation and thank the following reviewers for the time and dedication that they have shown *Pharmaceutics*:
Aamir JalilKyuichi KawabataAaron AlfordKyung-Oh DohAarti SharmaLacramioara PopaAbbas RahdarLaiba ShafiqueAbby C. CollierLalitha GummidiAbdelaziz ElgamouzLamar O. MairAbdelsamed I. ElshamyLamiaa M. A. AliAbderrahim YassarLan XiaoAbdollah KoolivandLana NežićAbdorreza Mohammadi NafchiLara BarazzuolAbdul Arif KhanLara Saftić MartinovićAbdul BasitLarisa A. TsarkovaAbdullah AssiriLarisa VolovaAbdullah IsrebLarissa BernardiAbdus SamadLarissa KotelevetsAbhilash SamykuttyLászló HazaiAbhimanyu DevLászló JicsinszkyAbhimanyu ThakurLászló SzilákAbhishek DeyLata SinghAbhishek JuluriLatifa Rbah-VidalAbhishek KumarLaura Carolina Zanetti-DominguesAbhishesh MehataLaura CatenacciAbílio SobralLaura D’AlfonsoAbshar HasanLaura FananiAbul Kalam AzadLaura K. S. ParnellAchraf NoureddineLaura MayolÁdám HaimhofferLaura MusazziÁdám JuhászLaura Oliveira-NascimentoAdam KlimowiczLaura RombolàAdam PacławskiLaura SbârceaAdam ReichLaura TrandafirAdam SchrumLaura VicenteAdam T. SuttonLaurel SillerudAdel Al-FateaseLauren M. HookAdéla MelcrováLaurent WillemsAdemar Alves Da Silva FilhoLavinia VlaiaAdeola O. AdebisiLaxmansa KatwaAdina Magdalena MusucLayla NabaiAdina Turcu-StiolicaLazaros I. SakkasAdina-Elena SegneanuLeandro Augusto CalixtoAditya SingarajuLeandro M. O. LourençoAdnan ShakoorLeandro MamoneAdrián MatencioLeda GuzmánAdrian OoLeena P. BharathAdrian P. WiegmansLehua QiAdriana CiurbaLei NieAdriana Corina HanganLei WangAdriana Cristina UrcanLelidis IoannisAdriana O. SantosLenaic LartigueAdriana SmarandacheLenka LanghansovaAdriana TrifanLenuta ProfireAdriano PiattelliLenuţa ŞutaAdriano TroiaLeny JoseAdrienne L. WatsonLeo FrkanecAfang ZhangLeon IslasAffiong IyireLeona GilbertAftab AlamLeonard Ionut AtanaseAfzal HussainLeonardo PaganiAfzal ShaikLeonor DelgadoAgata Antosik-RogóżLeslie Chavez-GalanAgata JanowskaLetao YangAgata PucekLetícia Scherer KoesterAgba SalmanLeto-Aikaterini TzivelekaAgnese GagliardiLetteria MinutoliAgnese MagnaniLeu-Wei LoAgnese PoLi LiAgnieszka ChwilkowskaLi LinAgnieszka CzapikLi WangAgnieszka Ewa Wia̧cekLi XiaorongAgnieszka Jagiello-GruszfeldLi YaoAgnieszka KarbownikLia Mara DiţuAgnieszka KicelLiane Isabel Ferreira MouraAgnieszka Lecka-AmbroziakLianet MonzoteAgnieszka Owczarczyk-SaczonekLiang MaAgnieszka RichertLiang Po HsiehAgnieszka Strzelecka-KiliszekLibo ZhangAgnieszka Swiatecka-UrbanLibor KostkaAgnieszka SzewczykLi-Ching ChangAgnieszka WnukLicia DossiAgostina LongoLidia GaffkeAgus LiandiLídia Maria Diogo GonçalvesAgustina NurcahyantiLidija GradišnikAhmad AbuhelwaLidwien Marieke HanffAhmad GholamiLifeng YanAhmad Ilyas RushdanLige TongguAhmad SalawiLígia N. M. RibeiroAhmed A. MohamedLihua ChenAhmed Abdallah HasanLi-Jun ChenAhmed AliLiliana ChemelloAhmed Al-KattanLiliana RodriguesAhmed BakryLilianne BeolaAhmed E. GodaLilith Caballero AguilarAhmed ElkamhawyLiliya S. LogoydaAhmed FatimiLina BaranauskieneAhmed GhallabLina Bezdetnaya-BolotineAhmed H. E. HassanLina DuAhmed H. El-GhorabLine Hagner NielsenAhmed I. KhodairLing WangAhmed M. Abu-DiefLing-Wen DingAhmed M. GoudaLingxiao XieAhmed M. MostafaLingyan ShiAhmed M. SayedLionel Fernel GamarraAhmed MostafaLipika ChablaniAhmed MoustafaLiron Limor IsraelAhmed N. AllamLisse Angarita DavilaAhmed NafisLiudmila MakarovaAhmed RagabLiudmyla V. GarmanchukAhmedova AnifeLivio RacanéAhmet KertmenLiviu DutaAhsan HameedLiwei YangAiden EblimitLiyuan ShengAilincai DanielaLjiljana DjekicAinhoa AlberroLloyd TangAiping LiuLluis LujanAishwarya IyerLluvia LópezAitana TamayoLong QueAiva PlotnieceLong-Sen ChangAi-Xiang DingLoredan Stefan NiculescuAjay Vikram SinghLoredana MarcuAjaybabu V. PobbatiLoredana UrsoAjeet KaushikLorena SegaleAkebe Luther King AbiaLorena ZentilinAkhilesh RaiLorenzo BonettiAkhtar NadhmanLorenzo Ferro DesideriAkihiko YamamotoLorenzo GuazzelliAkihisa OtakaLorenzo StellaAkihito HashidzumeLoris RizzelloAkilavalli NarasimhanLorne GolubAkira NakajimaLouis Kuoping ChaoÁkos JerzseleLuana PerioliAkriti SrivastavaLubov SnegurAkshaya BhagavathulaLuca FalzoneAlain E. Le FaouLuca FasolatoAlain Faivre-ChauvetLuca FilippiAlain-Pierre GadeauLuca GallelliAlan FairlambLuca MenichettiAlana ThackrayLuca PaluganAlberto BaldelliLuca PinziAlberto HidalgoLuca Salvatore De SantoAlberto Jorge Oliveira LopesLuca SoraciAlberto MarraLucas Bragança CarvalhoAlden EstepLucas D. DiasAldo Ferreira-HermosilloLucia BírošováAlejandro Pérez-LariosLucia CatucciAlejandro Romero MartínezLucia ČernákováAlejandro Samhan-AriasLucia De RosaAlejandro Schcolnik-CabreraLucia LopalcoAleksandar RaškovićLucia MontenegroAleksandar Ž. KostićLucia MorbidelliAleksander MendykLucia StančiakováAleksander VasilyevLucia ZakharovaAleksandr S. KazachenkoLucia ZemaAleksandra BukhovetsLuciana Pereira RangelAleksandra KlimczakLuciano PironeAleksandra KotyniaLucimara Gaziola de la TorreAleksandra M. BondžićLucinda V. ReisAleksandra MarciniakLúcio FrigoAleksandra NešićLucjan JerzykiewiczAleksandra Piechota-PolanczykLucjusz ZaprutkoAleksandra Popov AleksandrovLudmila Otilia CintezaAleksandra Sałagacka-KubiakLudmila V. PuchkovaAleksei A. StepanenkoLudwig CardonAlena FurdovaLuelak LomlimAlena Opálková ŠiškováLuigi BennardoAlessandra AmmazzalorsoLuigi MessoriAlessandra BigiLuigi MinafraAlessandra MaroniLuis Alberto Gaitán-CepedaAlessandra PisciottaLuis Angel Medina-JuarezAlessandra QuartaLuis ApoloniaAlessandra RossiLuis Eduardo DíazAlessandra ZilliLuis Enrique GomezAlessandra ZizzariLuis Exequiel IbarraAlessandro CeliLuis Jesús Villarreal-GómezAlessandro DalpiazLuís Pinto Da SilvaAlessandro GranitoLuisa RicciardiAlessandro GrattoniLuise LopesAlessandro MattinaLuiz Felipe Domingues PasseroAlessandro ParodiLuiza C. S. ErthalAlessandro PratesiLuiza Campos ReisAlessandro RizzoLukas HegerAlessia FazioLukasz CwiklikAlessia FilipponeŁukasz DobrekAlessio BocediŁukasz FijałkowskiAlexander A. Yu. Vasil’kovŁukasz SobottaAlexander A. ZhgunŁukasz ŚwiątekAlexander B TeslerŁukasz SzczukowskiAlexander E. BerezinŁukasz SzeleszczukAlexander FominLuminita CrisanAlexander KonevLuminita MarinAlexander KyrychenkoLuqin SiAlexander LjubimovLuz Kelly AnzolaAlexander MarinMaarten OomsAlexander NovikovMaciej J. KotowskiAlexander P. VoroninMaciej JaskiewiczAlexander SamokhvalovMaciej PrzybyłekAlexander ShpakovMaciej WłodarczykAlexander ShtilMadalin-Marius MarganAlexander SolmsMadan Lal VermaAlexander TsouknidasMadaswamy S. MuthuAlexandr Alexandrovich KolobovMaddeboina KrishnaiahAlexandra BarganMadhulika PradhanAlexandra BocsikMagali Benjamim De AraújoAlexandra Cristina MunteanuMagdalena Cristina StanciuAlexandra GaubertMagdalena HurkaczAlexandra ZamboulisMagdalena Jastrzębska-WięsekAlexandre VetcherMagdalena KotańskaAlexandrina NanMagdalena Krajewska-WłodarczykAlexandru GrumezescuMagdalena Lebiedzinska-ArciszewskaAlexey A. IvanovMagdalena Makarska-BialokozAlexey ChubarovMagdalena MatczukAlexey MikhaylovMagdalena OćwiejaAlexey NazarovMagdalena Paczkowska-WalendowskaAlexey S. VasilchenkoMagdalena PeruzynskaAlexey V. FeofanovMagdalena StaszczakAlexis BouinMagdalene J. SeilerAlexis OlivaMaged E. MohamedAlfio BattiatoMaha Saber-AyadAlfonso ZambonMahbubul MatinAlfred UltschMahdi RoohnikanAlfredo Téllez ValenciaMahendra DeonarainAli Akbar AshkarranMahima SinghAli Athab Al-kinaniMahmood AhmedAli BahadurMahmoud A. A. IbrahimAli FayazMahmoud BalbaaAli ImranMahmoud ElsabahyAli KhatibiMahmoud ElsayedAli MarufMahmoud El-ShahatAli TaghizadehMahmoud GhomiAli ZarrabiMahwash MukhtarAliakbar AshkarranMainul HaqueAlice ChateauMaja KatalinićAlice MelocchiMaja MatulićAlicia RovoMaja Ortner HadžiabdićAlicja KluczykMajell E. LaneAlicja Maria Sochaj-GregorczykMajella LaneAlicja Stachelska-WierzchowskaMajid Rasool KamliAlicja UrbaniakMajid Sakhi JabirAlicja Utrata-WesołekMakoni PedzisaiAlin CiobicăMakoto NishizukaAlin Laurentiu TatuMaksim RebezovAlina Crenguța NicolaeMaksym M. FizerAlina Gabriela RusuMakusu TsutsuiAlina Pyka-PająkMalavolta LucianaAline Yen Ling WangMalček MichalAlini Tinoco FricksMalgorzata BurekAlireza ZarebidokiMałgorzata JanickaAlisha PrasadMałgorzata JeleńAlla B. BucharskayaMałgorzata KujawskaAllen E. HaddrellMałgorzata MiastkowskaAloisio AnnamariaMałgorzata SznitowskaÁlvaro MeanaMałgorzata ZakrzewskaÁlvaro Zubizarreta-MachoMalik AydinAmaç Fatih TuyunMallesh KurakulaAman Ullah KhanMamoru TanakaAmanda Carroll-PortilloMamta ShahAmanda SiegelMandy BeyerAmany Mohammed FekryManindra TiwariAmeeduzzafar ZafarManish Vijaya KumarAmélia M. SilvaManja KurečičAmelia Ramon-LopezManju Rawat SinghAmin Jafari SojahroodManjunath Patel Gowdru ChandrashekarappaAmin ShavandiManoj Kumar TembhreAmir ElaloufManomi PereraAmirali PopatManoshi GayenAmjad KhanMansoore EsmailiAmmad Ahmad FarooqiMantasha IdrisiAmmar Abdulrahman JairounMantosh Kumar SatapathyAmmar BaderManuel AurelianoAmos M. YinnonManuel Bueno-MartínezAmparo Sánchez NavarroManuel Córdoba-DiazAmr ElShaerManuel Jose LisAmr HefnawyManuel Marques FerreiraAmr S. Abu LilaManuel Sanchez-DiazAmrindra PalManuel SuredaAna AlcudiaManuela A. HoffmannAna Beatriz Morales CepedaManuela CalinAna Belén Jódar-ReyesManuela ChiperAna BorotaManuela CurcioAna Borrego-SánchezManuela GernertAna CaruntuMar Fernandez-GutierrezAna Catarina BaptistaMarc André LécuyerAna Catarina DuarteMarc C. A. StuartAna CazacuMarc D. BassonAna Claudia CarreiraMarc HuismanAna Claudia PavarinaMarc MarescaAna DekanicMarc MauryAna FigueirasMarc PoirotAna ForgiariniMarc SchneiderAna GarcíaMarc SturgillAna Gonzalez NoyaMarcel BermudezAna Henriques MotaMarcela De MoraesAna I. MatesanzMarcela SegundoAna Isabel Alvarez-MercadoMarcelle Silva-AbreuAna Isabel BarbosaMarcello CiaccioAna Isabel FernandesMarcelo CorreiaAna Isabel Fraguas-SánchezMarcin GierekAna Isabel Torres-SuárezMarcin OżarowskiAna JaklenecMarcin SkotnickiAna Lucía Morocho-JácomeMarcin SobczakAna M. Carmona-RibeiroMarcin ZiemniakAna Maria MuţMárcio F. SimãoAna Maria UdreaMarcio TieraAna Marta de MatosMarco CalabròAna MornarMarco CarmignaniAna Paula BassiMarco ContardiAna Paula CandiotaMarco CordaniAna PilipovicMarco CosentinoAna Podolski-RenićMarco D’AbramoAna PretoMarco GovoniAna ReyMarco LocatelliAna Rey RiosMarco MarcarelliAna Sara CordeiroMarco OrecchioniAna Vujačić NikezićMarco PaolettaAna ŽugićMarco PortelliAnaclet NgezahayoMarco SalvatoreAnahi Elena Ada BucchiniMarco SegattoAnaly Salles De Azevedo MeloMarco UboldiAnamaria BrozovicMarcos Augusto de Lima NobreAnamaria CristinaMarcos Martínez-GarcíaAna-Maria PutzMarcus Tullius ScottiAnandharajan RathinasabapathyMarek BrzezińskiAnanth Kumar KammalaMarek DrozdzikAnas AhmadMarek FolAnastasia GeorgiadiMarek K. DobkeAnastasia MpakaliMarek KonopAnastasia SpiliopoulouMarek LangnerAnastasia TsingotjidouMarek MuriasAnastasios GotziasMarek PagáčAnca Adriana ArbuneMarek PokornyAnca DinischiotuMarek PruszynskiAnca Giorgiana GrigorašMargareta Hammarlund-UdenaesAnca Maria JuncanMargarida Fernandes Baptista e SilvaAnca Niculina CadinoiuMargarita A. SazonovaAnca RoseanuMargarita E. NeganovaAnca StanaMargarita ParraAnca-Maria ArseniuMargarita TenopoulouAn-Chi TienMargherita PomattoAncy JosephMaría Adelina Jimenez ArellanesAnders ÖrbomMaría Ángeles PeinadoAndi AlijagićMaria Angeles RojoAndi Dian PermanaMaria Antònia BusquetsAndrás KorisMaria AtanassovaAndrás SzilágyiMaria Begoña Delgado-CharroAndraz DovnikMaria BonferoniAndré AraujoMária Budai-SzűcsAndré Ferreira MoreiraMaría C. ArufeAndré L. SimãoMaria C. TeixeiraAndré LuxenMaria Carmela Bonaccorsi Di PattiAndré Moreni LopesMaría Carmen MarquésAndré Rolim BabyMaria CristianoAndré SilvaMaria Cristina OliveiraAndrea ArsiccioMaria D. Rikkou-KalourkotiAndrea BernardosMaria de Gracia Garcia Martin Andrea ButeraMaria de las Mercedes Garcia-GilAndrea De PieriMaría Dea-AyuelaAndrea DefantMaria del Carmen Morán BadenasAndrea M. PotocnyMaría del Rocío Reyes MontesAndrea Maria GuarinoMaria DemeterAndrea MezzettaMaria DigiacomoAndrea PanunziMaria DoligalskaAndrea RagusaMaría Esperanza RuizAndrea RónaváriMaría Fernanda Montenegro-LandívarAndrea Roxanne J. AnasMaria FrantziAndrea VenerandoMaria Grazia BottoneAndrea ZanelloMaria Grazia OrtoreAndreas MelzerMaria Helena Andrade SantanaAndreea IacobMaria Helena FernandesAndrei HonciucMaría Hortigón-VinagreAndrei I. KhlebnikovMaria IgnatAndrei IvanetsMaría Isabel Rial-HermidaAndrei S. PotapovMaria IskraAndrei SurguchovMaria J. MouraAndrej PerdihMaría Jesus GarridoAndreja Leboš PavuncMaría Jesús Rodríguez-YoldiAndrés Da Silva-CandalMaria João RamalhoAndrés M. DurantiniMaria Jose MorillaAndrew D. GreenhalghMaria Julia AltubeAndrew HungMaria KalivaAndrew KatsifisMaria KallergiAndrew PoveyMaría L. Flores-LópezAndrew R. BurgoyneMaria LaptevaAndrew ShelenkovMaria LazaridouAndrew T. LucasMaria Leonor FaleiroAndrew UrquhartMaría Luisa MoyáAndrew V. HealyMaria Luisa TorreAndrey I. Poddel’skyMaria MaaresAndrey P. AnisimovMaria Manuela GasparAndrey SudarikovMaria Margarida CardosoAndrey Zamyatnin Jr.Maria MirabelliAndriy VoronovMaría MorosAndrzej CzyrskiMaria Natalia CalienniAndrzej K. CiechanowiczMaria NikolovaAndrzej SlominskiMaria PapasavvaAndrzej SwinarewMaria Pia FerrazAneesh KoyappayilMaria PratAneliya MilanovaMaria Principia ScavoAneta NowakiewiczMaria Rosa GigliobiancoAneta Ostróżka-CieślikMaria RuzzeneAngel Guillermo BracamonteMaría Socorro Espuelas MillánAngel León-BuitimeaMaria StrianeseÁngel Luis García-OtínMaria Teresa MasucciAngela AbruzzoMaria VittinghoffAngela CapocefaloMaría Vivero-LopezAngela ChemelliMaria WallertAngela CorvinoMariadoss Arokia Vijaya AnandAngela IvaskMaria-Jose Garcia FernandezAngela Patricia HernandezMarialuigia FantacuzziÂngela SousaMarialuisa MennaAngela SpoialăMariamena ArbitrioAngela StaicuMarián PeciarÁngeles JuarranzMariana MatiasAngelina AngelovaMariana PintealaAngélique SourMariana S. CastroAngelo FacchianoMariann HarangiAngelo TagliettiMarianna BarbalinardoAnibal De Freitas Santos JuniorMarianna PatrauchanAnicuta StoicaMarianne PrévôtAnil K. YadavMariano García-ArranzAnil VangalaMariano LicciardiAnisha D’SouzaMariano Martínez-VázquezAnişoara CîmpeanMariano VenanziAnita BakraniaMariarosaria MilosoAnita CohenMaricarmen Recillas-MotaAnita HafnerMarie Arsenian HenrikssonAnjan MotamarryMarie-Alix PoulAnkana KakotiMarie-Christine JonesAnkit GangradeMarie-Christine OnutaAnkit K. RochaniMariella TeránAnkit SanejaMarieta ConstantinAnkit TanwarMarija Gavrovic-JankulovicAnkur SharmaMarija IvanovAnkur SoodMarija LesjakAnkur VaidyaMarija M. BabićAnmol KumarMarijana Radić StojkovićAnn SimpsonMarika PellegriniAnna A. EfimovaMarilena Antunes-RicardoAnna Angela BarbaMarin MicutzAnna BelcarzMarina DukhinovaAnna BeniniMarina FialloAnna BiernasiukMarina FilimonovaAnna BirkováMarina KostićAnna ChoromanskaMarina KrjuchkovaAnna CieślińskaMarina Maria BelletAnna Cleta CroceMarina NisnevitchAnna Czajkowska-KośnikMarina RautenbachAnna Di SalleMarina S. VasilyevaAnna DrabczykMarina SalaAnna E. GullerMarina V. DunaevaAnna FilipekMario D’AcuntoAnna JelińskaMario FruschelliAnna KleczkaMario PetrettaAnna LechanteurMário R. C. SoromenhoAnna Lucia TorneselloMario RaspantiAnna MassaguerMário RosadoAnna MichopoulouMario Vincenzo RussoAnna MielańczykMario ZorattiAnna NegroniMarioara AvramAnna NowakMariola KozłowskaAnna PerriMarios G. KrokidisAnna ProcopioMarios SpanakisAnna Rita BiliaMarique AucampAnna ScomparinMaris LaubertsAnna SobańskaMarisa ColoneAnna StavitskayaMaristella MaggiAnna V. VologzhaninaMariusz KepczynskiAnna Wiktorowska-OwczarekMariusz NiemczykAnna WilkaniecMariusz SikoraAnna Winiarska-MieczanMariusz UchmanAnna ZaninoniMarjan SalariAnna-Liisa KuboMarjorie JonesAnnaluisa MaricondaMark C. HowellAnnamaria PallagMark GoldAnnarita FalangaMark JaroszeskiAnnarita StringaroMark McLaughlinAnne HulinMark R. PrausnitzAnne SeidlitzMark RinnerthalerAnne-Grete MärtsonMarko KrstićAnne-Marie CaminadeMarko MilerAnne-Marie Pensé-LhéritierMarko MilojevićAnoop KumarMarko SeverAnoop RawatMarlene SantosAnouk MullerMarloes Dekker NitertAnroop NairMarlon Henrique CardosoAnselm JordaMarta De ZottiAntero J. AbrunhosaMarta GalloAnthony D. M. CurtisMarta GomarascaAnthony DelalandeMarta González-ÁlvarezAnthony HarkerMarta Karaźniewicz-ŁadaAnthony K. L. LeungMarta KepinskaAnthony William ColemanMarta KotAntoine KichlerMarta LaranjeiraAnton FicaiMarta Martínez-AlonsoAnton J. M. LoonenMarta Michalska-SionkowskaAnton LarenkovMarta Miotke-WasilczykAnton P. TyurinMarta MonzónAnton SimeonovMarta PutrinšAnton TyurinMarta S. CarvalhoAnton V. KiselevMarta SzekalskaAntonella CasiraghiMarta Ziegler-BorowskaAntonella RivaMartha Cecilia Rosales-Hernández Antonella RosiMartin A. MasuelliAntonella SgarbossaMartin B. DornerAntonella SmeriglioMartin G. O’TooleAntonello A. BarresiMartin GoslingAntonello Di PaoloMartin GustavssonAntonello SantiniMartin KuentzAntoni Llopis-LorenteMartin PéčAntonia MancusoMartin S. StaegeAntonia ResslerMartin ŠímaAntonio Bernabé-AntonioMartin StimpfelAntonio Boix-MontañésMartin VlkAntonio Casado-DíazMartina BergantAntonio Di MartinoMartina KonerackáAntonio Di StefanoMartina PeršeAntonio FerreiraMartina RossiAntonio FronteraMarwa Ahmed SallamAntonio G. SolimandoMarxa L. FigueiredoAntonio GloriaMary Beth Browning MonroeAntônio J. RibeiroMary Cano-SarabiaAntonio Mario BulfamanteMaryam A. Shetab BoushehriAntonio MassaMaryam ArdalanAntonio Palumbo PiccionelloMaryam Borhani-HaghighiAntonio Romero-EstradaMaryam DaneshpourAntonio Romo-MancillasMaryam Mobed-MiremadiAntonio RussoMaryna StasevychAntonio Simone LaganàMarzena JamrógiewiczAntonio Topete CamachoMarzia VasarriAntonio Victor Campos CoelhoMarzieh SalimiAntonio ZuccaMasahiko SugimotoAntonios E. KoutelidakisMasahiko YamaguchiAntoon J. M. LigtenbergMasahito ShimojoAnu AiraksinenMasakazu SugishimaAnuj GargMasaki MorishitaAnuj KumarMasami OkamotoAnupam DhasmanaMasamitsu KanadaAnuraag BoddupalliMasanobu KomatsuAnvarhusein IsabMasanori AritaAnzhelika VorobyevaMasatoshi KarashimaApostolos PapachristosMasatoshi OhnishiAppu Vengattoor RaghuMasayuki YamamotoApurba Krishna BhattacharjeeMasood Alam KhanAraceli Castillo-RomeroMasrina Binti Mohd NadzirArafa MusaMassimiliano PerducaAránzazu Zarzuelo CastañedaMassimiliano TognoliniArash RamedaniMassimo ConeseArasu GanesanMassimo GulisanoArchana AkalkotkarMassimo LazzariAri MeersonMassimo TacchiniAriane RozzaMassimo ValotiArianna Carolina RosaMastoura Mohamed EdreesAriel Colaço De OliveiraMateja J. PrimožičArif KhanMateusz GliwińskiArif LuqmanMateusz KurekArijit NathMatilde Duran LobatoArindam PramanikMatja ZalarAri-Pekka KoivistoMatjaž FinšgarAristeidis ChiotellisMats RembergerAristeidis DokoumetzidisMatteo D’amoreArjun H. BanskotaMatteo Di GiosiaArkadi ArinsteinMatteo MoriArkadij SobolevMatteo MozzicafreddoArkadiusz JózefczakMatteo PaciniArkadiusz MatwijczukMatthew DonaduArkadiusz OrchelMatthew T. WolfArkadiusz SzpicerMatthias KönigArkadiusz ZarzyckiMatthias M. HerthArman Moini JazaniMatthias WackerArmando Pérez TorresMattia TiboniArmandodoriano BiancoMauricio Comas-GarciaArnaud BertschMauricio Moncada-BasualtoArno KwadeMauricio MoraisArno WieheMaurizio AlimandiAroa Tardáguila-GarcíaMaurizio GabrielliÁrpád TósakiMauro BancheroArpana VaniyaMauro CivesArpita ChatterjeeMauro MalvèArshad MahmoodMauro PistelloArtem D. RogachevMauro ViganòArthi ShridharMax BastianArthur RobertsMax KurlbaumArtur Ga̧dekMax LeongArtur GiełdońMax PiffouxArtur RibeiroMaxim PuchkovArtur ŚwierczekMaximiliano Luis CacicedoArtur Tadeusz KrzyżakMaya DymovaArtur TurekMaya MascarenhasArturo Edgar Zenteno-GalindoMaya ZaharievaArun ButreddyMayank PatelArun Kumar Narayanan NairMayank SinghArun Kumar TeotiaMayukh GuhaArunas StirkeMayur K. LadumorArunkumar RengarajMazen NazalArvin HonariMd Abdul HakimArvind GulbakeMd Abdus SubhanArvind NegiMd Habban AkhterArvind Singh ChandelMd Jamal UddinArwyn Tomos JonesMd Masud ParvezArzu AriMd Shahid SarwarAsad UllahMd Shakhawath HossainAsako YamayoshiMd. Amdadul HuqAshang LaivaMd. FaiyazuddinAshish AgrawalMd. Imtaiyaz HassanAshish NoronhaMd. JakariaAshkan BighamMd. Khalid AnwerAshlee D. BrunaughMd. RizwanullahAshok K. ShakyaMeda-Lavinia NegrutiuAshok KakkarMegan A. WaldropAshraf N. AbdallaMehdi Arbabi GhahroudiAsif NawazMehdi Shafiee ArdestaniAsim Ur. RehmanMehnaz KamalAsiya R. MustafinaMei-Chin LuAsmaa KhafagaMei-Hwa LeeAsta JuzenieneMelania BabincovaAsteria Luzardo-ÁlvarezMelgardt De VilliersAsterios PispasMelinda KolcsárAstrid BuicaMelis OlcumAswani Dutt VadlapudiMelvin Anyasi AmbeleAtchudan RajiMeng WuAtefeh ZarepourMengxi ShenAtheer AwadMengxing LiAthina AngelopoulouMeong Cheol ShinAthina PyrpasopoulouMepur RavindranathAthri D. RathnayakeMercedes FernandezAtiah H. AlmalkiMercedes Jiménez RosadoAtiđa SelmaniMetin UzAtsushi B. TsujiMh Busra FauziAtsushi KambayashiMia SivenAttila FarkasMiaorong YuAurelian Cristian BoscorneaMicael HardyAurélie SchoubbenMicaela B. ReddyAurélien DeniaudMichael A. FirerAurelio LunaMichael ArkasAurica P. ChiriacMichael DanquahAurica PrecupasMichael GrayAurore DanigoMichael GütschowAvathamsa AthirasalaMichael HofmannAvi DombMichael J. W. JohnstonAvneesh KumarMichael LeitgesAxel G. GriesbeckMichael M. GottesmanAxel H. SchönthalMichael MildnerAyman El-FahamMichael WelshAysegül AksanMichael WoeltjeAyuob AghanejadMichail KastelloriziosAzat BilalovMichal AlexovičAzhar RasulMichal CichomskiB. NagarajaganeshMichał KulusBabak BakhshinejadMichał PikułaBabur Z. ChowdhryMichal SmahelBahijja Raimi-AbrahamMichal SobkowskiBal L. Lokeshwar Michał Stefan LachBanzeer Ahsan AbbasiMichal ZarobkiewiczBao HongliangMichel PfefferBaolin GuoMichel RayarBarbara BiondiMichel VouéBarbara Blanco-FernandezMichela CastagnaBarbara C. KahlMichela FerrucciBarbara HajdukMichela GeminianiBarbara HausottMichele De LucaBarbara MalawskaMichele Di CosolaBarbara MavroidiMichele GalluccioBarbara MulloyMichele Georges IssaBarbara PavanMichele SavianoBarbara R. ConwayMichele SchlichBarbara SartoriMichitaka OzakiBarbara TomaselloMicol Silic-BenussiBarbara WicherMie KristensenBarkat KhanMiguel A. García-SánchezBart De GeestMiguel A. Tejada GiráldezBartlomiej KalaskaMiguel Armengot-CarcellerBartosz TrzaskowskiMiguel Aythami Ayuso SebastianBasanth Babu EedaraMiguel Gisbert-GarzaránBasheer MannasahebMiguel IdoateBasil MathewMiguel QuilianoBaskaran InbarajMiguel Rebollo-HernanzBassam AyoubMiguel SantosBassant BarakatMiguel Toro GonzalezBeata Hukowska-SzematowiczMiguel ViñasBeata Morak-MłodawskaMihaela BalasBeata Mrozikiewicz-RakowskaMihaela DeaconuBeata OlejnickaMihaela GhicaBeata PajakMihaela TrifBeata StaniszMihai Cosmin CenariuBeata Szulc-MusiołMihail MitovBeata ŻołnowskaMihail Virgil BoldeanuBeatriz Godínez-ChaparroMihalj PoŝaBeatriz Hernández-CarlosMika O. RuonalaBeatriz LimaMike DoddBehnam SadeghiMikhail G. AkimovBei ChengMikhail KryuchkovBéla KovácsMikhail V. KhvostovBelén AltavaMikhail V. ShestakovBelén BeginesMikhail Y. InyushinBelen Perez-SolansMikihito KajiyaBen J. BoydMila LovrićBeng Fye LauMilad SheydaeiBenjamí Oller-SalviaMilan GautamBenjamin B. KastenMilan JakubekBenjamin CaroneMilan MladenovićBenjámin GyarmatiMilan VranesBenjamin HeymanMilena A. VegaBenjamin JurekMilena VukicBenjamin Le OuayMilica AćimovićBenno WeigmannMilica BorovcaninBenny RyplidaMilica LukicBenoit DenizotMilica MarkovicBenqing ZhouMilica PantićBeom-Jin LeeMiljan MilosevicBeom-Jun KimMilka MilevaBerenice Fernández-RojasMilo GattiBerna Sarıyar AkbulutMiloš D. MiljkovićBernadette-Emőke TelekyMilos KojicBernard DoMin Hua ChenBernd RathkeMing ChenBernhard SpinglerMing Hsien ChanBettina SchwarzingerMing-Ching HsiehBettina WollankeMingfu ChenBetty Yuen-Kwan LawMingjun TsaiBetül Arıca-YeginMing-Kuem LinBeverley D. GlassMingsheng ChenBhagyashree JoshiMinna M. HankaniemiBhasker RadaramMinyahil WolduBhupal P. BhetwalMiodrag JanićBhupendra PrajapatiMiran SebestjenBhushan MunjalMircea TampaBhuvana Shyam DoddapaneniMireia MallandrichBiagio RaponeMirela MihailaBikash R. PattnaikMireya De La GarzaBiliana NikolovaMiriam HickeyBiljana ArsicMirko ManchiaBiljana MojsoskaMiroslava A. NedyalkovaBilqees BanoMirosław AniołBin ChenMiroslaw JanowskiBin LiMirta MilićBin LiuMiryam Criado-GonzalezBindu SubhadraMititelu Tartau LilianaBing HanMitja KrižmanBing ZhouMitra AliabouzarBingde ZhengMitsutaka KitamuraBingmei FuMladena Lalic-PopovicBini GeorgeMoamen S. RefatBinita ShresthaModestas ŽiliusBirgit GlasmacherModesto De CandiaBirgit HabedankMoema HausenBissera PilichevaMohamad AlamehBiswajit BhattacharyaMohamad YasminBiswajita PradhanMohamed A. A. OrabiBjoern BauerMohamed A. AbdelgawadBlanca Elí Ocampo-GarcíaMohamed A. GoudaBlanca Hernández-LedesmaMohamed A. HassanBlanca Lorenzo VeigaMohamed A. M. El GendyBlanca Ocampo-GarcíaMohamed Abd ElkodousBłażej PoźniakMohamed AddiBo DongMohamed Al-FatimiBo SunMohamed AwadBo WangMohamed El RaeyBo XiaoMohamed El-AassarBo ZhangMohamed HaiderBoaz MoharMohamed M. RadwanBogdan Ionel TambaMohamed Osman RadwanBoguang YangMohamed S. NafieBor Luen TangMohamed SalehBoris ErshovMohamed SallaBoris TenchovMohamed ShaabanBorislav AngelovMohamed SharafeldinBoya LiuMohamed W. AttwaBożena KarolewiczMohamed ZoughaibBożena Pawlikowska-PawlęgaMohamed-Elamir F. HegazyBozena TyliszczakMohammad A. AzadBożena ZgardzińskaMohammad A. SaadBozhao LiMohammad Abdul WahabBrad BarnettMohammad Akhlaquer RahmanBradley S. FergusonMohammad Al-HatamlehBrajesh SinghMohammad Amin MoosaviBrandt BertrandMohammad Amjad KamalBranislav StankovicMohammad AshfaqBrendan M. DugganMohammad Azam AnsariBrendan McMorranMohammad H. AlyamiBrendan T. GriffinMohammad HashempurBreogan Pato DoldanMohammad HaskaliBrian CicaliMohammad IkramBrian D. AdamsMohammad Javed AnsariBrígida Ribeiro PinhoMohammad MominBrijesh M. ShahMohammad NajlahBrittany G. SemanMohammad Rahimi-GorjiBrun PaolaMohammad Reza MozafariBruna LarattaMohammad ShahidBruno Bueno-SilvaMohammad Tavakkoli YarakiBruno F. O. NascimentoMohammad Yaseen AbbasBruno Henrique VilsinskiMohammad Zaki AhmadBruno TassoMohammadreza ShokouhimehrBryan TroxellMohammed E. ElsalantyBryan WongMohammed ElmowafyBryn D. MonneryMohammed JafarBuck RogersMohammed Qasim ChaudhryBuddhadev LayekMohan Kumar Muthu KaruppanBulent AhishaliMohandoss SonaimuthuBulmaro CisnerosMohd Abul KalamBurak TüzünMohd Ahmar RaufBurcu GumuscuMohd Saeed SaeedByeongmoon JeongMohd. Zaheen HassanByeong-Ui MoonMohit GuptaByungsuk KwonMohsen AfounaCaijun SunMojca BozicCaisheng WuMojca LunderCalas André-GuilhemMojtaba VaismoradiCale D. FahrenholtzMominur RahmanCalogero FioricaMona I. ShaabanCamelia Sorina StancuMona K. E. QushawyCamelia UngureanuMónia A. R. MartinsCamilla FogedMónica BenitoCamilla MatassiniMonica CiveraCamilo Zamora-LedezmaMonica MariniCândida TomázMónica TéllezCanyang ZhangMonika A. OlszewskaCara-lynne SchengrundMonika CzerwinskaCarina CruchoMonika JaneczkoCarla M. LopesMónika KiricsiCarla Marcella CaramellaMonika Musiał-KulikCarla MucignatMonika Śmiga-MatuszowiczCarla PalumboMonique DeonCarla PollastroMonirosadat SadatiCarla RozzoMontserrat GarcíaCarla SardoMoriah KattCarlo GalliMorito KurataCarlo IraceMorteza KafshgariCarlo VecchioMorvarid KabirCarlos Alfonso Tovilla-ZarateMoshi GesoCarlos Alonso EscuderoMostafa BaghaniCarlos Alonso-MorenoMostafa MabroukCarlos CaroMotoki OkamotoCarlos David Grande TovarMotoki UedaCarlos Fernando MourãoMouhamad KhoderCarlos J. CarrascoMR Naimi-JamalCarlos Torrado-SalmerónMubashar RehmanCarlos Torres-TorresMudit TyagiCarlota O. Rangel-YaguiMuhammad AkhtarCarlotta FigliolaMuhammad AyyoobCarlotta PontremoliMuhammad Hammad ButtCarmen De KockMuhammad Imran RahimCarmen DinizMuhammad JunaidCarmen FerreroMuhammad Mustafa AbeerCarmen Gutierez-MillanMuhammad N. MahmoodCarmen RinaldiMuhammad NawazCarmen ScholzMuhammad OvaisCarmen WänglerMuhammad QasimCarmine ColucciniMuhammad RasoolCarmine OnofrilloMuhammad Shahid IqbalCarmine OstacoloMuhammad Sohail ZafarCarol BarryMuhammad UmairCarolina de Aguiar FerreiraMuhammad UmerCarolina EcheverryMuhammad Usman GhoriCarolina MarquesMuhammad Usman MinhasCarolina SerenaMuhammad Usman MunirCaroline SubraMuhammad Wahab AmjadCarolyn M PrimusMuhammad ZamanCarson J. BrunsMuhammad ZubairCarsten GrötzingerMuhammad Zubair IqbalCatalina Natalia Cheaburu-YilmazMumuni MomohCatarina I. V. RamosMuralimohan CheepuCatarina RosadoMurtaza M. TambuwalaCatarina SeabraMurugesan ChandrasekaranCaterina GuiotMutian HuaCaterina LiciniMuzaffar IqbalCaterina PalleriaMuzamil KhatriCatherine TuleuMuzamil WantCátia DominguesMyeong-Hyeon WangCécile VignalMykola IsaievCecilia MariniMyoung-Hwan ParkCecilia Matilde Fernández PonceMyron R. SzewczukCécilia Ménard-MoyonNada M. MostafaCecilia Zavala-TecuapetlaNader ShehataCédric HubertNadezhda GolubkinaCédric OrelleNadia NajafiCélia Tavares de SousaNadir BettacheCeline HenoumontNadja EngelCélio Geraldo Freire-de-LimaNae Gyu KangCerasela Elena GîrdNagavendra KommineniCésar de Julián FernándezNageswari YarravarapuCesar SaldiasNahed A. HussienCesare CamettiNahuel OlaizCetin CanpolatNaidu SeetalaChabaco ArmijosNan HaoChaiyavat ChaiyasutNan WangChalermpong SaenjumNancy Monroy-JaramilloChanchal Deep KaurNancy NisticòChandra KothapalliNanda Gopal SahooChandrabose SelvarajNanda MandavaChandrashekhar VoshavarNan-Hung HsiehChang ChiannNaoaki SakataChang-Hyun OhNaohisa OgoChangqing SuNaoki YoshikawaChangseon RyuNapaphak JaipakdeeChang-Sik HaNarendar DudhipalaChang-Tong YangNaresh KasojuChangwei WeiNaresh Kumar RajendranChangwon YangNarissara LailerdChanin SillapachaiyapornNarsimha MamidiChao LiNashiru BillaChao QiNasim ChiniforushChao ZhangNasim NosoudiChaoliang HeNatalia A. MuralevaChariklia Sotiriou-LeventisNatalia KrastevaCharkoftaki GeorgiaNatalia KulesskayaCharles AffourtitNatalia L. KlyachkoCharles TruilletNatalia LinkovaCharng-Cherng ChyauNatalia ShnayderCharvi NanavatiNatália TomašovičováChatchakorn EurtivongNatalia Wawrusiewicz-KurylonekChathura AbeywickramaNatalie FilmannCheang KhooNatalie L. ListerChen TanNatallia V. DubashynskayaChendong HanNatasa MilosevicCheng HuNataša NastićCheng-I LeeNatasa SiminCheng-Kang ChiangNatasa Skalko-BasnetCheng-Liang HuangNatassa PippaChengpeng FanNathalie Heuzé-Vourc’hChengyue WuNathan K. EvansonChengzhong YuNathan SeligsonChenyu LiNavaneet ChaturvediCheol Gi KimNaveed AhmadCheol HwangboNaveen ChellaCheol MoonNaveen MekalaChi Chun WongNaveen RaghavendraChia-Hao SuNavid RabieeChia-Hung ChouNawaf Al-MaharikChia-Hung LeeNayan Ranjan SinghaChi-An ChengNazira El-HageChiara BrulloNebojša PavlovićChiara Da PieveNectarios VidakisChiara MartinelliNedasadat Saadati ArdestaniChie KojimaNeelesh Kumar MehraChieh-Cheng HuangNeeraj Kumar SethiyaChien Ing YeoNeeraj ThakurChien-Feng LiNeil McgregorChien-Ming HsiehNeil StrotmanChih-Kuang ChenNelson GomesChih-Li LinNelson OssesChi-Ming WongNemany HanafyChin-Chean WongNemat AliChinghsiung LinNenad D. FilipovićChinh Tran To SuNenad FilipovicChin-Ping KungNenad IgnjatovicChin-Yu LinNenad JoksimovićChiranjeevi KorupalliNermeen Adel ElkasabgyChi-Ting HorngNeşe Buket AksuChitra ThakurNeslihan Üstündaǧ OkurChiung-Hui LiuNesrein HashemChi-Yun WangNéstor Mendoza-MuñozChristel NeutNgbolua Koto-te-NyiwaChristian Erick PalavecinoNgu TrinhChristian Espinosa-BustosNguyen ThanhChristian LuebbertNguyen Tien HuyChristian PaetzNiall M. H. McLeodChristian WölkNicholas G. FischerChristina Cortez-JugoNicholas PeakeChristina KaravasiliNicholas SmithChristina N. BantiNicola IngramChristina TangNicola OreficeChristine DufèsNicola OrzalesiChristine HappelNicola PuglieseChristine WhiteNicola SalvareseChristoph AlexiouNicola VanacoreChristoph PortierNicolae BacalbaşaChristophe CurtiNicolas AbatzoglouChristophe PadoinNicolas MongardonChristopher AsquithNicolas PottierChristopher F. AdamsNicolas TournierChristopher LucasNicole C. L. NoelChristopher RobertsNicoleta Nicole RaduChristos G. TakoudisNidhish FrancisChristos I. GioumouxouzisNidia M. León-SicairosChryssa BekiariNiels Frimodt-MøllerChuan ChenNiels RöckendorfChuanming XuNihal S MuliaChuanqi PengNikesh GuptaChuda ChittasuphoNikita TsvetovChulhun ParkNiklas SandlerChun Hung ChuNikola AnđelićChun Nam LokNikola StefanovićChun XuNikolaos BouropoulosChun Yuen ChowNikolaos NazirisChung Hang ChoiNikolaos TsetsosChung-Jung LiuNikolay A. PyataevChung-Ping YuNikolay O. Mchedlov-PetrossyanChung-Sung LeeNikolay PolyakovChunhong DongNikolett Kállai-SzabóChunjung ChenNikos Ch. KarayiannisChunsheng XiaoNikunjkumar PatelChunyang GuNikunjkumar VisaveliyaCiara O’FlanaganNil PandeyCinzia Anna VenturaNilakshi BaruaCinzia PaganoNina BonoCiprian DanielescuNina FilipczakClara GrossoNing LiClara Isabel Colino GandarillasNing ZhangClara López-IglesiasNinganagouda PatilClara RàfolsNir QvitClare HoskinsNisar Ul KhaliqClassius Ferreira Da SilvaNishad Thamban ChandrikaClaudia CavaNishant KulkarniClaudia Daniele Carvalho NavarroNishant S. KulkarniClaudia DettoriNitesh K. KundaClaudia DeusNitin CharbeClaudia DurantiNivedita ShettyClaudia KitzmüllerNobuhito HamanoCláudia Maria F. PereiraNoelia González-BallesterosCláudia P. PassosNoelle S. WilliamsClaudia PigliacelliNoemí EncinasClaudia ScottiNoha AttiaClaudio BucoloNongyue HeClaudio CaprariNoor AlmandilClaudio de LuciaNootan PandeyClaudio FerranteNoppawan MoralesClaudio LuchiniNor Eddine SounniClaudio MaiaNora KlötingClaudio SalomónNorazlinaliza SalimClaudio SorioNorhan Abd El-AzizClaudio Téllez SotoNoriaki IijimaClivel CharltonNoriaki NagaiCodruta SoicaNoriko SatoCody PeerNorio SogawaConggang ZhangNorma Sofía Torres PabónConstain H. SalamancaNorrie PearceConstantin MircioiuNuno Hélder SilvaConstantin NechitaNuno R. FerreiraConstantinos TsioutisNuno ValeConsuelo CelestiNunzio DenoraConxita MestresNur Najmi Mohamad AnuarCooper ItzikNurhasni HasanCoralia BleotuNuria AlegretCornel BaltăNuttawut SermsathanasawadiCosima HirschbergOana Andreea CojocaruCosmin Mihai VesaOana CadarCostas KiparissidesOana Catalina MocioiuCostica CaizerOana Viorela NistorCotas JoãoObaid AfzalCraig A. McElroyObinna ObianomCraig BlackstoneOctavian Tudorel OlaruCrispin DassOkhil NagCristian MateiOkky Dwichandra PutraCristian MunteanuOlaf SommerburgCristian O. SalasOleg LevinCristian PeptuOleg V. GradovCristian Sandoval Olena IvashchenkoCristian ScheauOlena TaratulaCristiana GonçalvesOlexandr S. ZaichenkoCristiane Aguiar Da CostaOlga A. KostCristiane Damas GilOlga AvrutinaCristiano DiasOlga LuzinaCristian-Tudor MateaOlga MusskayaCristina AlonsoOlga MysiukiewiczCristina BarthaOlga TarasovaCristina ChircovOlga UrbanekCristina FornagueraOlga V. GrinevaCristina GhiciucOlga V. KarpovaCristina GiannattasioOlga YarovayaCristina MaccalliniOlgica NedicCristina Manuela DrăgoiOliver ThewsCristina MarchiniOlivera Mitrović AjtićCristina MartinezOlivia DorneanuCristina Martin-SabrosoOliviu VostinaruCristina Mihaela NicolescuOluwaseun F. EgbelowoCristina MüllerOluwatoyin A. AdelekeCristina NistorOmar BreikCristina PadulaOmar MertinsCristina PuigjanerOmar SarheedCristina QuílezOmer AydinCristina SatrianoOndřej KostovCristina TubaroOndřej SlanařCristina VercelliOndrej VasicekČrtomir PodlipnikOnofrio LaselvaCsaba HetényiOrapan PoachanukoonCsilla Özvegy-LaczkaOsagiede OsayandeCsilla SzebenyiOsama MadkhaliCustodia FonsecaOscar Marcos-ContrerasCyril FersingOsmar Antonio Jaramillo-MoralesCyrille RichardOtilia CostisorCyrille SabotOvidio CatanzanoCyrus MotamedOwen KavanaghCzeslaw RadziejewskiOyuna KozhevnikovaDa SunOzgul GokDaan J. A. CrommelinPablo BotellaDae Hwan ShinPaiboon JungsuwadeeDae Won ChoPalanisamy NallasamyDaiqin ChenPalaniyandi KaruppaiyaDaiva BironaitéPalash SanphuiDali ZhengPall Thor IngvarssonDalibor ŠatínskýPálma FehérDamian PiotrowskiPaloma De La Torre-IglesiasDamiana Téllez-MartínezPamali FonsekaDana AkilbekovaPanagiotis BarmpalexisDana NiculaePanagiotis KaranisDana ZahaPanagiotis PeitsidisDanail PavlovPanoraia I. SiafakaDancho DanalevPanteleimon E. PapakonstantinouDandan GuoPao-Chu WuDanica MichaličkováPaola CartaDaniel Andrés RealPaola ChiodelliDaniel ArcanjoPaola FacherisDaniel BaeckerPaola FerrariDaniel Fernández-QuirozPaola FiniDaniel Fernández-VillaPaola LaurienzoDaniel FologeaPaola LenziDaniel González-NietoPaola MelottiDaniel H. González MaglioPaola StoriciDaniel JancuraPaola TaddeiDaniel Martinez-MarquezPaolo FagoneDániel NemesPaolo GiunchediDaniel NeureiterPaolo GovernaDaniel P. OttoPaolo M. ScriminDaniel PotaczekPaolo MarianiDaniel SchramekPaolo PellegrinoDaniel SitarPaolo TrucilloDaniel SmržPapović SnežanaDaniel Vasile BalabanParaskevi G. MitliangaDaniel W. NixonParijat KanaujiaDaniel ZakowieckiParisa MojaverDaniela CalinaParise AdadiDaniela Cristina CulitaParthasakha DasDaniela Di GiuseppeParthiban VenkatesanDaniela IannazzoPascal A. Le CorreDaniela MarascoPasquale ScarciaDaniela MiricescuPatial VikramDaniela MontiPatrice A. MarchandDaniela RusuPatrícia AlvesDaniela SuzukiPatricia BogdanovDaniela Vieira BuchaimPatricia Gálvez-MartínDanijela PetrovicPatricia Garcia-LopezDanilo Martins Dos SantosPatrícia M. ReisDanina KrajišnikPatricia P. MartinsDanish IqbalPatrícia Ribeiro PereiraDanuta BranowskaPatricia SeverinoDanuta Januszkiewicz-LewandowskaPatrícia SeverinoDaocheng WuPatricio Iturriaga-VasquezDaodao HuPatrick AnglardDaozong XiaPatrick KoczeraDapeng ChenPatrick PoucheretDaria PoshinaPatrick QuelemesDarija Cör AndrejčPatrick T. RonaldsonDarin ZertiPatrick-Julian MesterDario BragaPatrizia ChetoniDario MizrachiPatrizia FerroniDariusz Maciej PisklakPatrizia Nadia HaniehDariusz T. MlynarczykPatrizia Polverino de LauretoDarya RadziukPatrizia ZaramellaDashevskiy AndriyPatrycja Domalik-PyzikDavid BerlanaPatrycja KleczkowskaDavid BraydenPaul BaroneDavid Chávez-FloresPaul Cătălin BalaureDavid DolivoPaul Chi Lui HoDavid DrewryPaul DalhaimerDavid FitzGeraldPaul G. LayerDavid GerberPaul JoyceDavid GreenhalghPaul MatejtschukDavid HaložanPaul MozdziakDavid HercherPaul RöschDavid J. LundyPaul RoyallDavid JonesPaul Seke EtetDavid LandyPaula CampelloDavid LimónPaula CoutinhoDavid MillsPaula FerreiraDavid OrlikowskiPaula M. T. FerreiraDavid StewartPaula OrtegaDavid Vicente-ZurdoPaula OssowiczDavide FirinuPaula PintoDavide Giuseppe RibaldonePaula SchaiquevichDavood KharaghaniPaulina CeglaDawei ZhaoPaulina Cortes-HernandezDawid PrzystupskiPaulina KazimierczakDayun YanPaulius RuzgysDe DasguptaPaulo Cesar De MoraisDebabrata MandalPaulo Jorge PalmaDebika DattaPaulo MatafomeDeblina BiswasPaulo PaixãoDebora CarpanesePavel KopelDebora Decote-RicardoPavel MadonovDebora FerreiraPavel RozsivalDeborah A. LanniganPavel SolopovDeck Khong TanPavol RajniakDeepak KulkarniPawan MishraDeependra Kumar SinghPaweł ChmielarzDeependra SinghPaweł CichoszDeepika RaiPawel Dabrowski-TumanskiDelia Ioana HorhatPaweł KafarskiDelia Mihaela RaţăPaweł KwiatkowskiDelia MunteanPaweł LenartowiczDemba SarrPawel NakielskiDeng-Guang YuPawel OlczykDenis ButusovPaweł Piotr PomastowskiDenis RychkovPawin PongkorpsakolDenitsa B. MomekovaPedro AmorósDennis AdjepongPedro BarataDennis DouroumisPedro DoradoDer Yuan ChenPedro FonteDerek SmolenskiPedro Mendoza De GivesDerya AytemizPegi Ahlin GrabnarDésirée WünschPei Yuin KengDetlef NeumannPei-Ling HsiehDevapriya SinhaPeiyuan WangDevendra KumarPelin Erkoc ErikDevika ChithraniPeng ChenDevis BenfaremoPeng GaoDezhuang YePeng QuanDhanasekaran VikramanPeng XueDharamdeep JainPeng ZengDharmendra Kumar YadavPeng Zou (China)Dhirender KattiPeng Zou (USA)Diana Andreia Tavares PintoPer HanssonDiana Camelia NuţǎPer LarssonDiana Drago-GarcíaPeta HarveyDiana Elena CiolacuPetar PetrovDiana Marcela Aragon NovoaPetar TodorovDiana MostafaPeter GalettisDiana NicaPeter HauserDiana PortanPeter N. LipkeDiana RafaelPeter NeyerDiana SavuPeter O’ConnellDianbao ZhangPeter WaldeDianjun CaoPetr FilipDibakar DharaPetr ZamostnyDibya PandaPetra KolencDidier DesmaëlePetra KosutovaDiego Romano PerinelliPetra KotnikDiego TesauroPetra MajerovaDijendra Nath RoyPetra WiseDimitrios BikiarisPetras PrakasDimitrios G. FatourosPetre IonitaDimitrios GkiliopoulosPeyman GholamiDimitrios RekkasPhatsawee JansookDimitrios SklirosPhei Er SawDimitrios TsiourvasPhil AyersDimitris KardassisPhil BormanDimitris StellasPhilip ChennellDina CoertzenPhilip SarajlicDinesh DhumalPhilip WertzDinesh KumarPhilippe BarthélémyDinesh Kumar VermaPhilippe BillialdDinesh NyavanandiPhilippe BuletDinesh UpadhyaPhilippe CauchieDionysia PapagiannopoulouPhilippe CotelleDipankar RoyPhilippe LesotDirga LamichhanePhilippe-Henri SecretanDivya SharmaPhitchayapak WintachaiDjordje MedarevicPhiwayinkosi V. DludlaDmitriy AtochinPhuong-Hien NguyenDmitriy SmolenskyPier Giorgio CojuttiDmitry A. StetsenkoPiera Di MartinoDmitry A. TikhonovPierre Alexandre KaminskiDmitry E. PostnovPierre Le BarsDmitry KostyushevPierre RocheteauDmitry VerbenkoPierre TufferyDmytro DmytriievPierre-Antoine BonnetDolores Gutierrez-AlanisPierre-Louis ToutainDolores R. SerranoPierre-Simon BellayeDomagoj GlavinaPierre-Yves Collart-DutilleulDomenico AlbanoPietro CacialliDomenico FrancoPietro CalissanoDomenico TricaricoPilaiwanwadee HutamekalinDominik SelzerPilar López-CornejoDominik WöllPınar ŞenDominique LesuissePing SongDonald TomaliaPing YaoDonatella ArmentanoPing YipDonatella Degl’InnocentiPing-Chung LeungDonatella PontiPin-Kuang LaiDonatella TaramelliPio ContiDonato BonifaziPiotr DobrzynskiDonato ColangeloPiotr GarnuszekDonato Di VenerePiotr KawczakDonato SantovitoPiotr KulinowskiDong ChenPiotr KurcokDong Hee NaPiotr MuchaDong Hyun LeePiotr OkińczycDong LiangPiotr SzwedaDong Wuk KimPiroska Szabó-RévészDongfang ZhouPiyush GuptaDonghyun LeePiyush MehtaDongin KimPlamen KatsarovDong-Kyu KimPlamen KirilovDongrim SeolPo-Huang LiangDong-Seol LeePolina A. DeminaDongwei ZhangPoliraju KalluruDong-Woo ChoPompilia Ispas-SzaboDong-Wook HanPoorva SandleshDongyang ZhangPorfiryevaDongyue XinPoushali DasDorian MigońPrabakaran NagarajanDorin DragosPrabhakar BusaDoris RušićPrabhanjan GiramDorota ChudobaPradeep KumarDorota DanielakPradeep Kumar BollaDorota Haznar-GarbaczPradeep Kumar PandaDorota SulejczakPradeep VaradwajDorota WarmińskaPraful R. NairDorota Wójcik-PastuszkaPrakash GangadaranDorothee WeihrauchPrakash RaiDouglas HooperPrakash ThapaDragana GabrićPranay SrivastavaDragana Mitić-ĆulafićPranothi MulintiDragana Vasic AnicijevicPrasanth ManoharDragana VasiljevićPrasenjit MondalDragos HorvathPrashant KumarDrenka TrivanovicPrashanth NageshDuane Choquesillo-LazartePrasopchai PatrojanasophonDuarte De Melo-DiogoPrasun KumarDulari JayawardenaPrathap SomuDuo ZhangPrathyusha BagamDurga Kalyani PaturiPratibha KumariDušan MalenovPraveen P. Nekkar RaoDwaipayan MukherjeePreeti ChhabraDylan PeukertPrem Pratap SinghE. Scott SillsPriscila Dauros SingorenkoEcevit BilgiliPriscilla AmaralEckart HanekePriya BharateEder Lilia RomeroPriya MurugasenEdgar Julian Paredes-GameroPriyadarshi ChakrabortyEdgardo DurantiniPriyanka BhattEdina RusenPriyanka RayEdio MaldonadoPriyanka ShawEdit CsapóProfeta MartinaEdmond GravelPrzemysław DorożyńskiEdoardo SinibaldiPrzemysław KołodziejEdorta Santos-VizcainoPrzemysŁaw ł. ZalewskiEdson C. Silva-FihoPrzemyslaw SiejakEduardo CostaPui Shan ChowEduardo Ferreira MolinaPuneet KhandelwalEduardo Fuentes QuinterosPunit P. SethEduardo Gutiérrez-AbejónPushpa Raj JoshiEduardo Maffud CilliPuting DongEduardo Montoya-DiazPyung-Hwan KimEduardo Padilla CamberosQaisar MaqboolEduardo SavioQamar AhmedEdurne GonzálezQi CaiEdward B. NeufeldQi ChenEdward J. PavlikQi GaoEdward J. SayersQi JinEdward RenQi ShaoEdward StephensQian YangEdyta BiskupQiang LiuEdyta Gendaszewska-DarmachQiang ShiEdyta MakuchQiangzhe ZhangEero VasarQian-Xiong ZhouEgidio BarbiQin FuEgor OsidakQing ChenEhab TahaQingzhen YangEhsan KarimiQinxue HuEhsan MahdiniaQiubai LiEiichi SatoQiuliyang YuEiji KubotaQonita Kurnia AnjaniEiji YubaQuanying BaoEirini ChristodoulouQubo ZhuEishi AshiharaQuentin VanhaelenEitoyo KokubuQun ZhouEkaterina A. LitvinovaRabijit DuttaEkaterina NaumenkoRachel SmithElaheh AllahyariRadoslaw DrozdElaine ChowRadosław KujawskiEleftherios G. AndriotisRadosław StarostaElena AznarRadostina GeorgievaElena BoggioRadovan BilekElena BoldyrevaRadu AlbulescuElena CicheroRadu C. RacovitaElena De MattiaRadu Claudiu FierascuElena DreassiRadu MineaElena I. RyabchikovaRafael Contreras-CaceresElena L. VodovozovaRafael DavalosElena NikolskayaRafael G. Campos-MontielElena PincuRafael Prado-GotorElena TchetinaRafael R. CastilloElena UspenskayaRafael Scaf De MolonElena V. KudryashovaRafał BielasElena V. ParfenyukRaffaella SoletiElena V. SvirshchevskayaRaghvendra A. BoharaElena VismaraRagna BerthelsenElena ZelepugaRaha OrfaliEleni KaratzaRaimar LobenbergEleni PapanikolaouRaimundo Fernandes De Araújo JúniorEleonora CasagniRainer TietzeEleonora MarettiRaj KumarEleonora NapoliRaj MukherjeeEleonora RussoRaj YadavEleonore FröhlichRajakumar GovindasamyElham AhmadianRajasekhara NerimetlaElham PishavarRajmohan DharmarajElhissi AbdelbaryRakesh BhaskarElia BariRakesh KhilwaniElia RanzatoRakesh TiwariEliana Aparecida de Rezende DuekRalf KircheisEliana LeoRaluca EftimieElias ChristoforidesRaluca IanchisElie H. MotulskyRaluca NicuElina Vuorimaa-LaukkanenRam Kumar SelvarajuElio PizzoRama BansilElisa BelluzziRamachandran VinayagamElisa PanzariniRamapuram Jayachandra BabuElisabete M. S. CastanheiraRamar ThangamElisabetta CanettaRamasamy PaulmuruganElisabetta EspositoRamendra Pati PandeyElisabetta GaviniRamesh MukkamalaElisabetta GrilloRamiro Guerrero-SantosElisabetta OrlandiniRamiro Palazón-GarcíaEliza WolskaRamon EritjaElizabeth VafiadakiRamón Martínez-MáñezElka TouitouRamón Mauricio Coral-VázquezEllas SpyratouRamón Peña-GarciaElliot V. HershRamón Sotomayor-ZárateElquio Eleamen OliveiraRamona D’AmicoEl-Sayed R. El-SayedRamya Lakshmi RajendranElwira ChrobakRanhua XiongElwira SieniawskaRania M. HathoutElżbieta BudziszRanjan RamasamyElżbieta PoniewierkaRanjit DeElżbieta WyskaRanjith Kumar RajamaniEman AshourRaquel De Cássia Dos SantosEman AtefRaquel De M. BarbosaEman MazyedRaquel Fernández GarcíaEman MehannaRaquel PetrilliEmanuele GiurisatoRaquibul HasanEmanuele PriolaRathapon AsasutjaritEmel BalogluRatnakar TripathiEmi HaladjovaRaúl Cazorla LunaEmili BesalúRaul G. EnriquezEmilia FisicaroRavi Chakra TuragaEmilia Iglesias MartínezRavi Kumar CheedaralaEmilia Janiszewska-TurakRavinder KumarEmilia MarcheiRavindra ThakkarEmilia TojoRavindranadh KoutavarapuEmilia TomaszewskaRaviraj VankayalaEmiliano ManzoRay W. BasrowiEmilie ReberRaymond C. C. WongEmilio ParisiniRayudika Aprilia Patindra PurbaEmily CaponeRazique AnwerEmma Adriana OzonRebeca AlvariñoEmma HughesRebeka RudolfEmma PiacentiniReda F. M. ElshaarawyEmmanuel A. PilaRegina KasperekEmmanuel Silva MarinhoRegina PutsEmőke Margit RédaiRégis GuieuEnass A. Abdel-HameedReham HamidaEngy ElekhnawyReham WasfiEnrica ChiesaReinier HernandezEnrico FerrariRéka BarabásEnrico GugliandoloRekha M. Ramesan Enrico IaccinoRemi VenezianoEnrico MoroRemy PoupotEnrique NizaRenata Salgado FernandesEoghan Joseph MulhollandRenata Vidor ContriEric AbenojarRenátó KovácsEric ChappelRenato LeoneEric HuetRené HuberEric J. MunsonRené KöffelEric S. HoRene St-ArnaudEric YagerRenjith JohnsonErica SmearmanRezvan JamaledinErik AarntzenRicardo MallaviaErik LauriniRicardo Nalda-MolinaErika A EksiogluRicardo Teresa RibeiroErika Bevilaqua RangelRiccardo Di CoratoErin W. HowardRichard D’ArcyErnandes Tenório-NetoRichard HorniblowErnesto Beltrán-PartidaRidhima JunejaErnesto GargiuloRiham Adel TawfikErnesto ReverchonRiku KawasakiErshuai ZhangRipon SarkarEsmeralda DelgadoRishi ThakkarEssa SaiedRisto KoivulaEstela Noguera-OrtegaRita CasadioEsther MorenoRita HumeniukEstuardo Lopez-VeraRitesh RamdhaniEthan LippmannRitu GuptaEthel J. NgenRiyaz Ali OsmaniEugenia BezirtzoglouRizwan WahabEugenia Rita LaurianoRob KeyzersEugenio ProvenzanoRobert BrinsonEugenio R. MéndezRobert CzarnotaEun Seong LeeRobert DickinsonEun-Ju KoRobert E. StratfordEurico LimaRobert GriffinEuzébio Guimarães BarbosaRobert J. MeierEva BernalRobert J. NashEva Fischer-FodorRobert LindnerEva Maria SantosRobert O. WilliamsEva NovotnáRobert R. BiesÉva SzökőRobert StrickleyÉva Zámboriné NémethRobert ZymlińskiEvangelos AxiotisRoberta MoschiniEvelyne Jacqz-AigrainRoberto De LucaEvgenia MitsouRoberto GramignoliEvgeny E. BezsonovRoberto MacovezEwa Kijeńska-GawrońskaRoberto MandrioliEwa LaskowskaRoberto NigroEwa M. UrbańskaRoberto PaciottiEwa Ostrowska-LigȩzaRoberto PasseraEwa Stodolak-ZychRoberto PisanoEwelina DziurkowskaRoberto RozasEyal RosenRobin CurtisEzzat KhanRocco Di GirolamoFabian I. EzemaRocío LitránFabian MohrRodica OlarFabiana GeraciRodica Paula TurcuFabiano VisentinRodolfo IppolitiFabio GanazzoliRodolphe CarpentierFabio GasparriRodrigo Corrêa BassoFabio PastorinoRodrigo FerreiraFabio SciubbaRoger M. LeBlancFabio ZobiRogerio R. Sotelo-MundoFabricio Maestá BezerraRohit P. DugarFabrizio BertelloniRohitash JamwalFabrizio CalderaRok FinkFabrizio CartaRoksana MarkiewiczFabrizio Dal PiazRoland BenzFabrizio FabriziRoland Hans StauberFabrizio FontanaRolf DanielsFadis F. MurzakhanovRomain EychenneFahad KhanRomain GuinamardFaiyaz ShakeelRoman A. ZinovkinFaizul AzamRoman B. LesykFakhar UddinRoman BlahetaFalko PippigRomána ZelkóFan ZhangRomeo RomagnoliFang Liu (China)Romeo T. CristinaFang Liu (UK)Romina PezzoliFang TongRon J. KeizerFang-Cheng LiangRonald B. MooreFarid MenaaRonald TaylorFarooq SyedRongjin SunFarshad Moradi KashkooliRonit SionovFaruk Fonthal RicoRony SegerFatemeh Kazemi-LomedashtRosa BellavitaFatemeh MovahediRosa HernándezFatemeh TabatabaieRosalba MansiFatima BentoRosalia Maria Cristina PellitteriFátima García-VillénRosalino Vázquez-LópezFatma M. MadyRosana Simón-VázquezFatmanur Tuǧcu-DemirözRosario BaroneFaustino MollinedoRosario González-MuñizFausto TucciRosario MareFederica BalzanoRosario OlivaFederica BrunoRosario SarabiaFederica Di SpiritoRosemeyre A. CordeiroFederica FacchinRoshan KatekarFederica FogacciRosica MinchevaFederica ManninoRosivaldo S BorgesFederica PisaneschiRossella LauranoFederica TonoloRostyslav StoikaFederico PercheRoxana Liana LucaciuFedor NovikovRuba IsmailFei FanRuben Hernandez-AlcocebaFei GuoRuben Manuel Luciano Colunga BiancatelliFei PengRubén Varela-FernándezFei XingRuby John AntoFei YanRuczyński JarosławFeifei YangRudra PangeniFeipeng YangRudy J. ValentineFelicia Gabriela GligorRui CostaFelisa CilurzoRui HuangFelix BroeckerRui VitorinoFelix DitzingerRuilong ShengFelix Javier Jiménez JiménezRuitu LyuFelix WiedmannRumiana TzonevaFelix YemanyiRumyana MarkovskaFengchao LangRuosen XieFengqing YangRupanjali PrasadFerdinando ChiaradonnaRupendra ShresthaFerdinando NicolettiRustem R. ZairovFerenc FenyvesiRuth PrasslFernanda BoniRuth Prieto-MonteroFernanda Nervo RaffinRuwan JayasingheFernando CalzadaRuža FrkanecFernando CardonaRyszard SmolarczykFernando De MoraSaba ShahinFernando Lopitz-OtsoaSabata MartinoFernando Notario-PérezSabine MazerbourgFernando PorcelliSabiruddin MirzaFernando RemiãoSabit HusseinFernão D. MagalhãesSabna KottaFevzi BardakciSabrina OliveiraFeyza F. DarendelilerSabyasachi ChakraborttyFilbert TotsinganSachiko HirobeFilipe Hobi Bordón SosaSachin Subhash SurwaseFilippo BasagniSachin ThakurFilippo FratiniSadaf GilaniFiore P. NicolettaSadaf JahanFioretta AsaroSadanand PandeyFiras KobeissySadia NoorFirdos Alam KhanSadri ZnaidiFiroz AkhterSagrario Martín-AragónFirzan NainuSahi JasminderFlávia Almada Do CarmoSai H. S. BodduFlavia ZacconiSaiyang ZhangFlavio RizzolioSajal ShrivastavaFleming RodriguezSaliha MoutaharrikFlor H. PujolSalvador GonzálezFlorence Djedaïni-PilardSalvatore MagazùFlorence SiepmannSalvatore ScaccoFlorenci V. GonzálezSalvatore ScolavinoFlorentina GolgoviciSamar MansourFlorentina Monica RadulySameer AwadFlorentina-Daniela MunteanuSameh A. AhmedFlorian OlivierSameh El SayedFlorian PlattenSamer EzziddinFlorian SlimanoSamet ÖzdemirFlorin BorcanSamia Aci-SècheFlorin OanceaSamina AlamFoteinos-Ioannis DimitrakopoulosSamir V. JenkinsFotis IliopoulosSamira MalekmohammadiFranc VrečerSamo LešnikFrancesc Rabanal AngladaSamuel DontaFrancesc Xavier AvilésSamuel E. AdunyahFrancesca AccioniSamuel E. SchrinerFrancesca AielloSamuel Martins SilvestreFrancesca BruzzeseSamuel SamnickFrancesca GadoSamuel YalkowskyFrancesca LorenzinSanam DolatiFrancesca MastrottoSanchita KunduFrancesca OrzanSanda AndreiFrancesca SciontiSanda BocaFrancesca TruzziSandeep Kumar Reddy AdenaFrancesca UcchedduSandile Phinda SongcaFrancesco AulicinoSandip Ashok SonarFrancesco Briatico-VangosaSandip S. ShindeFrancesco CacciolaSandor BenyheFrancesco FrancescoSandra AnjoFrancesco GiansantiSandra CecconiFrancesco InchingoloSandra CitiFrancesco LopezSandra DavernFrancesco MarongiuSandra DonniniFrancesco MilanoSandra García-GallegoFrancesco Paolo BusardòSandra GhelardoniFrancesco PezzellaSandra I. D. SimõesFrancesco ScavelloSandra N. PintoFrancesco TrottaSandra PetrovicFrancina MunellSandra PintoFrancine Johansson AzeredoSandra RebeloFrancisco A. Gómez-VeigaSandra SimõesFrancisco Abad SantosSandrine Cammas-MarionFrancisco Alexandrino JúniorSandrine HuclierFrancisco CarmonaSandro SonninoFrancisco EpeldeSandy EldridgeFrancisco Fernández-CamposSangbum ParkFrancisco Humberto Xavier-JuniorSangho RohFrancisco Javier Bejarano LuqueSangkil LeeFrancisco Javier Otero-EspinarSangmin LeeFrancisco José OstosSangram SamalFrancisco M. Martin-SaavedraSang-Sup LeeFrancisco Marlon Carneiro FeijóSanika SuvarnapathakiFrancisco Rodrigues PintoSanja J. ArmakovicFrancisco SolanoSanjana DayalFranciszek GłówkaSanjay AroraFranco CervellatiSanjib BhattacharyyaFranco H. FalconeSankar RenuFrançois GuérardSankarprasad BhuniyaFrançois-Xavier LegrandSanne Van LithFrank AboubakarSanta CirmiFrank CackowskiSantiago Daniel PalmaFrank KrauseSantiago Garcia-VallveFrank StahlSantiago Torrado-SantiagoFranz WorekSantosh BashyalFrédéric CoutantSantosh PeddiFrédéric TewesSan-Yuan ChenFrederico De SousaSara A. Abdel GaberFrederico PittellaSara De MartinFrederik DenormeSara FedericiFrédérique Megnin-ChanetSara M. RobledoFredric M. MengerSara MoradiFruzsina WalterSara NavaFuad AmeenSara S. ReisFulvio RattoSara SilvaFung-Fuh WongSarah AllegraFuyi WangSarkar M. Abe KawsarFwu-Long MiSarpietro Maria GraziaGabriel Del Río GuerraSashi DebnathGabriel G. De LimaSathish DharaniGabriel Luna-BarcenasSathish Sundar Dhilip KumarGabriel SamascaSatoshi KodairaGabriela DorciomanSatyavani KaliamurthiGabriela Dumitrita StanciuSaulius ŠatkauskasGabriela GrazianiSaurabh MishraGabriela RapeanuSawittree SahakijpijarnGabriela V. VochitaSawsan A. ZaitoneGabriela VlaseSayan GangulyGabriele CandianiScott McCombGabriele CarulloSean HynesGabriele CaviglioliSebastian GnatGabriele MulthoffSebastian SchloerGaetano MarvertiSebastian SchwamingerGajanan GhodakeSebastian ZahnreichGalina V. KurlyandskayaSebastiano IntagliataGammal MahrousSébastien GrégoireGaneshlenin KandasamySébastien PenninckxGang WangSébastien SchmittGang WeiSekar VijayakumarGarbine AguirreSenta KapnickGareth R. WilliamsSeok Ki ChoiGarima SharmaSerena MeravigliaGarry KerchSerena VeschiGary GellermanSergei TverdokhlebovGary McLeanSergey AdoninGary MossSergey FilippovGary R. MatyasSergey LevitskiiGaspare PalaiaSergey N. AdamovichGastone CastellaniSergey O. BachurinGaurav BairwaSergey SedyhGaurav PandeySergey TimofeevGautam PandeySergey UspenskiiGautam SinghviSergey VatsadzeGautham GampaSerghei CovantevGawęcki MaciejSergio AbbateGayong ShimSergio CortelazzoGeetha BollaSergio Crespo-GarciaGellért Balázs KarvalySergio Gómez-GrañaGema Martínez-NavarreteSergio Granados-PrincipalGennadiy A. KostinSérgio P. C. AlvesGeorg HöfnerSérgio SchenkmanGeorge DimitriadisSergiu CoseriGeorge J. DugbarteySerhat Sezai ÇiçekGeorge Mihai NitulescuSerkan YílmazGeorge SharbeenSeth PincusGeorge SpaethSeth T. HousmanGeorge VassilopoulosSeung-Chun ParkGeorgeta Hermina ConeacSeung-Yup KuGeorgia N. ValsamiSéverine BärGeorgiana NitulescuSeyed Khosrow TayebatiGeorgios BokiasSeyed Mohammad Ali Hosseini RadGeorgios D. PanosSeyyed Mojtaba MousaviGeorgios EleftheriadisShabirul HaqueGeorgios PampalakisShadab MdGeorgios ParaskevopoulosShadeed GadGera SoniaShadi HoushyarGerald B. KastingShadia A. ElsayedGerald PfefferShadma W. AbdulwahabGerard DumancasShah KhanGerardo Leyva-GómezShah ZamanGerasimenko Alexander YuryevichShahanshah KhanGergo TothShahinaze FouadGerhard FranzShahla MirzaeeiGerhard P. PüschelShahnaz MansouriGerry LushingtonShailendra B. TallapakaGert FrickerShakeel IjazGesmi MilcovichShakina Yesmin SimuGeta CârâcShamee BhattacharjeeGéza RegdonShamik MajumdarGhanashyam AcharyaShamir CassimGhanshyam Singh ChauhanShams TabrezGhasem SargaziShana StoddardGheorghe IliaShang JiaGheorghe NechiforShankali PradhanGhulam Abbas (Pakistan)Shankar Jaikishan EvaniGhulam Abbas (Sultanate of Oman)Shanmugavel ChinnathambiGiacomo Dal BelloShaobin WangGiacomo LuciShaobo RuanGianandrea PasquinelliShaojian HeGiang V. H. PhanShaojie ChenGianina DodiShaojie LiGianluca ManniShaokoon ChengGianluca ViscusiShaoyong LuGideon KerstenSharan BobbalaGiedrė KasparavičienėSharon Shui Yee LeungGiedrius KalesnykasShatil ShahriarGijung KwakShazia BanoGilbert KochShazid Md. SharkerGilles LemaîtreSheila SpadaGilles LemercierSheiliza CarmaliGiorgia UrbinatiSheng YinGiorgio FacchettiShengnian WangGiorgio ManginoShengshuai ShanGiovanna BaronSher Singh MeenaGiovanna CalabreseSherine N. KhattabGiovanna Della PortaShian-Ren LinGiovanna PontarinShiao-Wen TsaiGiovanna RassuShiba DandpatGiovanni CasiniShibi LikhiteGiovanni Codacci-PisanelliShichao DingGiovanni DamianiShigeki SetoGiovanni Di BernardoShigeru KawakamiGiovanni Luca CeresoliShih-Feng ChouGiovanni RaugeiShih-Jen LiuGiovanni SignoreShi-Jye ChuGiovanni TossettaShindu ThomasGiovanni VigliottaShing-Hwa LiuGirish PattappaShin-ichi HiranoGirolamo CasellaShinji KobuchiGirum GetachewShintaro FumotoGiulia BernardiniShintaro SukegawaGiulia Di PrimaShiro KoizumeGiulia VantiShirui MaoGiuliana CangemiShiv Dutt PurohitGiulio FracassoShiva PathakGiulio Petronio PetronioShiying YangGiulio SanciniShoko ItakuraGiuseppe CirilloShraddha ParateGiuseppe GalloShreesh Kumar OjhaGiuseppe LazzaraShrikanth GadadGiuseppe MurdacaShruthi ShanmukhaGiuseppe PizzolantiShruti KabadiGiuseppe TrapaniShu FangGiuseppe ZanottiShuang YuGiuseppina IoeleShubai LiuGiuseppina NoccaShubhmita BhatnagarGiuseppina SabatinoShu-Chen HsiehGiuseppina SandriShu-Chuan LiaoGiyoong TaeShuhei YamadaGizem BorShuhua BaiGlaucio Valdameri ValdameriShu-Hui ChenGloria Álvarez-SolaShuichi SetoguchiGloria Huerta-AngelesShu-Ling HsiehGloria ManzottiShunqing TangGobi Saravanan KaliarajShusaku HayashiGokhan BahceciogluShuxiong ChenGonzález-Torres MaykelShyam Kishor SahGonzalo CampilloShyam Kumar GudeyGonzalo ScaleseShyam S. SamantaGonzalo VillaverdeSibel İlbasmiş TamerGopakumar GopalakrishnanSibhghatulla ShaikhGopinath KrishnasamySiddharth S. MatikondaGopinathan JanarthananSigbjørn BerentsenGorazd PojeSigrid C. RobertsGordana Wozniak-KnoppSijin GuoGordon WinterSilvia BistiGoutam MukherjeeSilvia CarvalhoGovindasamy ManiSílvia Castro CoelhoGrant CaveSilvia De FranciaGrazia SerinoSilvia FranzèGrażyna Odrowąż-SypniewskaSilvia GrassilliGreg GormanSilvia MarchesanGreg M. ThurberSilvia TampucciGregg DeanSilvia TortorellaGregg HendersonSilvio AimeGregory AmidonSimin SharifiGregory CaputoSimion KreimerGregory PoonSimon Chi Chin ShiuGreta FaccioSimon CleggGrzegorz CieslarSimon SieberGrzegorz SchroederSimon TröderGrzegorz TrybekSimón Yobanny Reyes-LópezGrzegorz WegrzynSimon ŽakeljGuang J. ChoiSimona BadilescuGuang-Bo GeSimona ClichiciGuanghui ZhaoSimona FontanaGuangle NiuSimona MirelGuangze YangSimona SaponaraGuan-Jhong HuangSimona TavernaGuanjun YangSimone BattagliaGuendalina ZuccariSimone BrogiGuenka PetrovaSimone Di MiccoGuido MerlottiSimone OttonelloGuido PanzarasaSimonida TomicGuilherme M. GelfusoSin-Yeang TeowGuillaume CombesSipho MdandaGuillermo Cristian Guadalupe Martínez ÁvilaSirajul HaqGul ShahnazSiranjeevi NagarajGun Yong SungSiriporn OkonogiGunjae JeongSitanshu S. SinghGunjan AroraSitaraman KrishnanGunjan PurohitSiu Hong Dexter WongGunnel HalldénSiva Rama Raju KanumuriGuofu LiSiyi GuGuohui LiSławomir JakiełaGuoyu PanSławomir MilewskiGurjaspreet SinghSmita SalunkeGuru Raghavendra ValicherlaSmrithi PadmakumarGurusamy SaravanakumarSnehal RautGus RosaniaSnezana Ilic-StojanovicGustavo FernandesSobhi M. GomhaGutemberg AlvesSobia HalimGuzel ZiyatdinovaSobia NoreenGwang Hyeon EomSofia ChatziioannouGyörgy KeglevichSofia LimaGyörgy Tibor BaloghSofia MattssonGyudo LeeSofia MitakouHae Gyun LimSofia SoaresHai HuangSohel M. JuloviHaibing ZhouSoliman KhatibHaibo ZhouSolmaz Maleki DizajHaifei ZhangSonali NashineHaigang WuSong YangHaiming LuoSónia CarabineiroHaiping ZhuSonia LaneriHaiqing DongSonia MartinsHaisheng HeSônia N. BáoHai-Shu LinSonia PinhoHaisu ZengSonja VisentinHaiyan XuSoohyun KimHaiyuan ZhangSophia HatziantoniouHaizhen Andrew ZhongSorin AvramescuHakan BozdoğanSorina DinescuHalyna OharSorina SuarasanHamdy AbdelkaderSotiris AmillisHamed NosratiSotiris HadjikakouHamid IrannejadSoumen SahaHamid MerchantSoumya Rahima BenhabbourHamid VanaeiSoundarapandian KannanHanan M. ElnahasSourav BhattacharjeeHang Fai KwokSpiro KonstantinovHang QiSravan PatelHang YiSreekanth VedagopuramHanif HaidariSreeram UdayanHan-Jia LinSri Vishnu Kiran RompicharlaHan-Joo MaengSrinath PashikantiHan-Jun KimSrinivas AjjarapuHanna BandarenkaSrinivas MutalikHannah BatchelorSrinivas SistlaHans ZoellnerSrinivasan KrishnanHans-Christian SiebertSrinivasulu AitipamulaHany Hamdy ArabSriram VaidyanathanHao NieSrujan MarepallyHao ZhangStanislav PantelyushinHao-Jen HsuStanislaw OłdziejHarald KolmarStanisław WołowiecHarant HannaStanko SrcicHardik AminStavros MalamatarisHarini MohanramStavroula SofouHaroon KhanStefan BroerHasan Hüseyin DoǧanStefan D. MomčilovićHassan AlbarqiStefan WinterHassan AlmoazenStefan ZóradHassan GhonaimStefania CometaHassan Y. EbrahimStefania ContiHassan YousefniaStefania RacovitaHaw-Ming HuangStefania ZampattiHaya Lorberboum-GalskiStefano AlcaroHayate NakagawaStefano BorocciHea-Young ChoStefano CardeaHeba A. GadStefano EramoHeba ElsewedyStefano FaisHeba M. AboudStefano Francesco CrinòHeba SahyonStefano Girolamo PalazzoloHee Eun KangStefano LeporattiHee Y. KangStefano MarchesiHee-Joon KimȘtefan-Ovidiu DimaHeejun ParkStephan Joel ReshkinHeidi KidronStephan KrâhenbûhlHeiko HeerklotzStéphane ChabaudHeinrich HaasStephanie AndradeHelen HughesStephanie E. BarrettHelena Bustos-CruzStéphanie Leroy-LhezHelena KellyStephanie LongetHelena P. FelgueirasStephanie M. Dorta-EstremeraHélène CôtéStephen A. MorrisHelmut GreimStephen AdamsHelmut H. PopperStephen ChimHemanth GudapatiStephen HsuHend Abdel BarStephen R. TomlinHend AbdelhakimStephen ShrewsburyHeng Phon TooStephen YarwoodHengyi XuSteve PascoloHenrik LaurellSteve WiltonHenrike LucasSteven BatinovicHenrique FernandesSteven EsworthyHenry H. Y. TongSteven R. DuncanHerman E. PopeijusSteven SmithHerman J. WoerdenbagSteven WillowsHermínio P. DiogoStewart B. KirtonHermis IatrouStuart CretonHesham El-SeediSu Hyun HongHesham TawfeekSubhani M. OkarviHiba NatshehSubodh K. SamratHideaki FujiiSubrata DebHidefumi KasaiSucharat LimsitthichaikoonHidehiro UekusaSudarson Sekhar SinhaHidekazu KawashimaSudi PatelHideki TeraiSudip Kumar PattanayekHideo KatoSudip MondalHideo SuzukiSudip MukherjeeHideo WadaSudipta PanjaHideto FukushiSuhail MohdHideto TsujiSuhwan LeeHideyuki KanematsuSui Yung ChanHideyuki KawauchiSujan K. MondalHideyuki SatoSukhvinder SinghHiep Tuan TranSuman AlishettyHimanshu KathuriaSumati BhatiaHira FatimaSun Tae KimHiroaki WakimotoSunbin DengHiroki YokotaSung Giu JinHiromi SakaiSung Mun LeeHiromu KondoSungtae YangHiroshi UedaSunil GairolaHiroyoshi Y. TanakaSunil SinghHiroyuki TsuchiyaSupandeep Singh HallanHisako FujiharaSurajit BhattacharjyaHisayuki KomakiSurendra NimeshHisham Al-ObaidiSuresh Kumar KailasaHishinuma ShigeruSuresh ThanguduHitendra PatelSuresh ValiyaveettilHiu Chuen LokSuryadi IsmadjiHoang-Thanh LeSusan HawthorneHoch MatthiasSusana García-SilvaHo-Jin LeeSusana MaellaHongbo ChenSusanne Florin-MuschertHongKee SahSusanne RospleszczHong-Ki LeeSusheel Kumar NethiHonglin JinSushobhana BandyopadhyayHongran YinSusi BurgalassiHongwei ZhangSuyong ReHongyan LiSuzana M. AndradeHonorata KraskiewiczSuzuki ShugoHooman TorabiSven RutkowskiHoracio CabralSvetlana A. KonnovaHo-Seong SeoSvetlana A. SorokinaHossain Mohammad Arif UllahSvetlana AidagulovaHossam M. HassanSvetlana BatashevaHossein HosseinkhaniSvetlana SaikovaHossein ZarrinfarSvetlana SanterHouliang TangSvetlana Viktorovna DemyanenkoHristo SvilenovSvitlana KopylHsien-Ming LeeSvjetlana RausHsiu-Wen ChienSwami Vetha Berwin SinghHsuanping ChangSwaminathan RajaramanHsueh-Wei ChangSwarup RoyHsuheng YenSwati PundHua HuangSyed A. A. RizviHua JinSyed Ali Raza NaqviHuaizhe YuSyed ImamHualu ZhouSyed MahmoodHua-Tao XieSyed Mohd Danish RizviHubert Van den BerghSylwia BudzynskaHubert ZiółkowskiSylwia ŻołądekHudson C. PoloniniSzczepan ZapotocznyHugh J. ByrneSzilvia BerkóHugo FernandesSzymon SękowskiHugo Morales-RojasT. J. HollingsworthHugo RochaTadakazu KondoHui LiuTadeusz PrzybyłowskiHui SunTae-Jong YoonHui ZhuTaek LeeHuihui FanTaesup LeeHuirong LeTaher HatahetHuixian HongTahseen KamalHuiyi CaoTaia Abd El-MageedHumaira YasminTaiki MiyazawaHun LeeTakahiro SatoHung Huey-ShanTakashi AzumaHung-Chi ChengTakashi IwasakiHung-Tse HuangTakashi SaitoHung-Wei YangTakashi SatoHung-Yu HuangTakayasu MishimaHuolong LiuTakayuki FuruishiHusam YounesTakayuki NakagomiHwi-Yeol YunTakehiko GotohHye Jin YouTakehisa HanawaHyesun GwakTakeji Takamura-EnyaHyo-Eon JinTakenori HarunaHyun Sook HongTaketo UchiyamaHyunah ChoTakuichi SatoHyung-Soo HanTakumi IshizukaHyun-Jong ChoTakuya MaeyamaHyun-Ouk KimTamako HataI. G. TiganovaTamara KrajnovićIan S. BlagbroughTamara R. TodorovićIan WymanTamás FehérIbolya AndrásTamás SoványIbrahim A. NaguibTamis DarbreIdoia GallegoTangui BarréIfat Sher-RosenthalTanja IlićIftekhar MahmoodTanveer A. TabishIgnacio LeonTanveer A. WaniIgnazio RoppoloTanzila SabaIgor A. PrudovskyTao JinIgor A. ZolotukhinTao LongIgor BuzalewiczTao YiIgor KukhtevichTao ZhangIgor ShmarakovTaoufiq BenaliIgor SlivacTarun K. MandalIkram SghaierTarun KeswaniIkram Ullah KhanTasha M. Santiago-RodríguezIlaria Elena PalamàTatiana AstrelinaIlaria FlorisTatiana PereiraIlaria IacobucciTatiana V. PletenevaIlaria OttonelliTatjana P. StanojkovićIldiko BadeaTatjana StevićIldikó SzabóTatsuaki TagamiIliana IvanovaTatu RojalinIliana Medina-RamírezTatyana A. TimofeevaIlidio J. CorreiaTatyana I. ShabatinaIlker S. BayerTatyana KoutzarovaIlkka JulkunenTatyana V. AbramovaIlma NugrahaniTatyana V. VolkovaIlona OściłowskaTe I. LiuI-Lun HsinTed LakowskiIlya KhodovTejabhiram YadavalliIlya ShenderovichTeodora Alexa-StratulatImaël Henri Nestor BassoléTeresa Ayora-TalaveraIman KavianiniaTeresa CasimiroI-Ming ChuTeresa CastilloImran AliTeresa KowalskaImran SaleemTeresa M. R. MariaInaam NakchbandiTeresa Maria ViseuInbar SchlachetTeresa Zardán Gómez de la TorreInderbir SinghTereza S. MartinsInès Esma AchouriTerezinha de Jesus Andreoli PintoIngrid Fricke-GalindoTerje DoklandIn-hwan BaekTero JärvinenIn-Lu Amy LiuTerrence PivaInna Rabinovich-NikitinTeruo MurakamiIntan Rosalina SuhitoTetsuya MiyanoIoan MamaligaTetsuya OzekiIoana Adriana MateiTetsuya ToyonoIoana Cristina OlariuThaigarajan ParumasivamIoanina ParlatescuThais AlvesIoannis GrivasThaíse Gonçalves AraújoIoannis NikolakakisThaned PongjanyakulIoannis PartheniadisThangavel PonrasuIoannis PaterasThanongsaksrikul JeeraphongIoannis PirmettisThashree MarimuthuIoannis S. ChronakisThawatchai PhaechamudIoannis TsamesidisThazha P. PrakashIoannis ZachosThea MagroneIogann TolbatovTheodora Venera ApostolIolanda De MarcoThi Thao Linh NguyenIole VendittiThi Tuong Vy PhanIon MîndrilăThiagarajan MadheswaranIonela NicaThierry VandammeIonut LuchianThilini ThrimawithanaIonut-Cristian RaduThiruganesh RamasamyIosif MarincuThomas D. RoperIouri E. BorissevitchThomas DandekarIrena MaliszewskaThomas DavisIrene BonadiesThomas GoudoulasIrene Gómez CruzThomas M. S. ChangIrene Lara-SáezThomas SwiftIrene Martínez-MartínezThundon NgamprasertchaiIrina G. GazaryanTiago DiasIrina GladkikhTiago SantosIrina KufarevaTian JingzhiIrina M. Le-DeygenTianlong LiIrina NaletovaTianqing LiuIrina PopescuTianyu LiIrina V. SemenovaTianyu YanIrina VelikyanTibor KibediIrina А. LutsenkoTicuta Negreanu-PirjolIrina-Georgeta SufaruTifeng JiaoIris MinichmayrTim DargavilleIris UsachTim PagetIrlon FerreiraTimo L. M. Ten-HagenIrshad Ahmed HajamTimofei ZatsepinIruthaya Pandi Selestin RajaTimothy HeathIsabel Desgagné-PenixTimothy NguyenIsabella OrientiTimothy NottoliIsabella PanfoliTin Wui WongIsadore KanferTina McKayIseli NantesTing ZhongIsha TanejaTing-Shu WuIslam A. KhalilTingtao ChenIslam HusainTitus VlaseIsmael Darío BiancoTivadar FeczkóIsmaheel O. LawalTivadar KissIsra AliTiziano MarzoIsrael AlshanskiTofayel AhmedIsrafil KucukTom AnchordoquyIssa Sulaiman Al-AmriTomasz BlicharskiIstvan AntalTomasz BogielIstván BányaiTomasz GrabowskiIstván BudaiTomasz GubicaIstván KertészTomasz JelińskiIstvan LekliTomasz KloskowskiIstván VinczeTomasz KowalczykItai BenharTomasz KoziorItamar GonçalvesTomasz KubrakIuliana PopescuTomasz PańczykIurii S. StafeevTomasz Robert SosnowskiIvan Cruz-ChamorroTomasz TokarekIvan GitsovTomasz W. KaminskiIván José Galindo-CardielTomislav KizivatIván Martínez-ValbuenaTomohiro OsakiIvan PalaginTomokazu OhishiIvan R. QuevedoTomomi AkitaIvan S. Mfouo-TyngaTon J. A. LoontjensIvan SalamonTong DengIvan SavicToru MatsuiIvan StoikovToshi NagataIvan V. BogdanovToshihiko TashimaIvana AleksićToshihiro Sakurai SakuraiIvana CacciatoreToyofumi SuzukiIvana DelalleTracie L. FerreiraIvana JochmanováTristan M. SissungIvana PantelićTrong D. TranIvana Škugor RončevićTse-Ying LiuIvaylo DimitrovTsung-Rong KuoIvelina TsachevaTsuyoshi KimuraIveta BernatovaTsvetelina VelikovaIvette Buendía RoldánTu LeeIvica AvianiTumey NathanIvo B. RietveldTuru GáborIwona GolonkaTze Ning HiewIwona Inkielewicz-StepniakTzi Bun NgIwona ŁakomskaTzu-Chieh TangIwona ZarzykaTzu-Yu PengIzabela Muszalska-KolosUbaldo ArmatoJ. Michael SorrellUday BagaleJ. Stephen LodmellUdaya KhandavilliJacek BaniaUdayabhanu JammalamadakaJacek BoguckiUgo ConsoliJacek JemielityUgo De GraziaJacek MatysUgur CakilciogluJacek NyczUjjal Kumar BhawalJacek PiosikUlf AndereggJackie Tan-ShowyinUlises Páramo-GarcíaJacobo Hernández MontelongoUlrike RitzJacopo BurrelloUluvangada Thammaiah UthappaJacqueline CloosUlvi GursoyJacquelyn Ann BrownUmberto MusazziJacques TheronUmesh S DeshmukhJaehwi LeeUmile Gianfranco SpizzirriJae-Hyuck ShimUpendar Rao GollaJaeseong OhUpendra SinghJafar RezaieUrszula BazylinskaJagpreet SinghUrszula KarczmarczykJaime Amaya-FarfanUrszula NawrotJaime Santoyo-SalazarUwe LiebchenJake NeumannVáclav RancJakub MatusiakVadanasundari VedarethinamJakub SuchodolskiVadde RamuJakub SzlękVahid SerpooshanJakub ZdartaVahideh RabaniJakub ŻurawskiValdecir XimenesJames C. WaltonValentina AnutaJames ChowValentina CastelliJames R. SmithValentina DincaJames V. AquavellaValentina GrumezescuJames X. HartmannValentina Laghezza MasciJan A. A. M. KampsValentina NigroJan BiernatValentina OrlandoJan BilskiValentina PucaJan GajdziokValentina VassalloJan Henrik FinkeValeria AmbrogiJan JežekValeria ChionoJan KotekValeria De MatteisJán KozempelValeria De PasqualeJan Rijn ZeevaartValeria MarigoJan SteenekampValeria PanzettaJan TheysValerie CarabettaJana HorakovaValerie PetegniefJana Šic ŽlaburValério Monteiro-NetoJanaki IyerValerio VolianiJanet NaleValery ZinchenkoJanet ThompsonValeryia MikalayevaJangik Ike LeeValle PalomoJanka BábíčkováVan Tu DuongJános BorbélyVanda Isabel Roldão Canelas Vaz SerraJarmila JedelskáVanderlei Salvador BagnatoJarmila ZbytovskaVandita KakkarJaromír MyslivečekVanessa Gonzalez-CovarrubiasJaroslav KocisekVanessa Liévin-Le MoalJarosław J. PanekVanessa VieiraJarosław WaloryVanja TadićJasmina ŽivanovićVanya Nikolova MantarevaJason LimVarish AhmadJason M. RauceoVartika SrivastavaJason Thomas DuskeyVarun KushwahJaved AhmadVasantha-Srinivasan PrabhakaranJavier Linares-PasténVasco BrancoJavier MendicuteVasil D. SimeonovJavier NakamatsuVasiliki TsikourkitoudiJavier Ventura-JuárezVassilis J. DemopoulosJaya DantamVasudeva IyerJayabalan NirmalVasyl M. HaramusJayapal Reddy MallareddyVeera Venkat Shivaji Rama Rao EdupugantiJaydeep YadavVeerawat TeeranachaideekulJean-Francois ColomerVehary SakanyanJean-Francois Lauzon-JosetVeikko LinkoJean-Yves Le QuestelVenkatakrishnan RengarajanJeesu KimVenkataswarup TiriveedhiJeevithan ElangoVera L. M. SilvaJeffrey Bryant TraversVerena ZerbatoJeffrey L. MoranVeronica AranJeffry G. WeersVeronika HuntosovaJelena Antić-StankovićVesna RadojevićJelena DinićVibhu PrasadJelena DjurisVicent Sixte Safont VillarrealJelena LazicVictor Ivanovich SeledtsovJelena ParojčićVictor Kuz’minJen-Chang YangVictor LoschenovJenchih TsengVictor Mangas SanjuánJeng-Shiung JanVictor MidlejJennifer E. NormanVictor RevinJennifer F. MoffatVictor TapiasJennifer HulingVictoria Campos-PeñaJeong Hun ParkVictoria FabrisJeong Uk ChoiVictoria GrishkoJeong-Hwan KimVictoria KlangJeremy KirkVictoria O. ShipunovaJérôme AlsarrafVictoriya A. GeorgiyantsJerzy BeltowskiViet LeJerzy Jan JaroszewskiViet-Khoa Tran-NguyenJeske WalterVijay Sagar MadamsettyJessica BridouxVijay TomerJessica Kubicek-SutherlandVijayabhaskar VeeravalliJessica RosenholmVikas Anand SaharanJesús María Hernández RivasVikas KumarJesus Porcayo-CalderonVikash KumarJi Woong ChangVi-Khanh TruongJiajun ZhuVikram SarpeJiale YangViktor Korzhikov-VlakhJia-Ling RuanViktor ReshetnikovJianfang Feng Viktoria RajtukovaJiangeng HuangViktória VargaJianhong WangViliana Eduardova GuglevaJianhua ZhouVilma PetrikaiteJiann-Ruey HongVinaya BasavarajappaJianping LiuVinayak KhodadeJianqiang LiuVinayakumar RaviJianqin LuVincent HascallJianwen LuoVincent J. SmithJianxun DingVincent YeungJiapeng LiVincenzo De LeoJiashen LiVincenzo GuarinoJiawei WangVincenzo QuagliarielloJia-Yaw ChangVinit RajJie GaoVinoth RajendranJie TangVioleta NiculescuJie ZhuViorica PatruleaJierui YuViorica Railean-PlugaruJieyu ZuoVirag VasJim La ClairViral KansaraJimenez Carolina PalaciosVirender SinghJin BaiVirginia MerinoJin GaoVirginia PensabeneJin Hae KimVishnu RevuriJin MotoyanagiViswas Raja SolomonJin WangVitaliy V. KhutoryanskiyJin YuVítor H. AlvesJinbin XuVittoria Di MauroJinbo FeiVittorio FineschiJinchao LouVivek PuriJinchul KimVivek TrivediJing WangViviana De CaroJingan LiViviane GoncalvesJingen ZhuVlad T. PopaJingjing GaoVladimir ArionJingjing WuVladimir DobričićJinli WangVladimir DyakonovJinrong MaVladimir G. SergeyevJinshan GuoVladimir I. KalininJinsong HaoVladimir Igorevich MakarovJintae LeeVladimir JakovljevicJinxiang XiVladimir L. KolesnichenkoJiong ZhouVladimir Lucian EneJiri KroutilVladimir N. MorozovJisook MoonVladimir TemchuraJiunn-Wang LiaoVladimir V. BezuglovJiwei GuVladimir V. ZarubaevJi-Young ParkVladimir YudinJoachim VenzmerVladislav FomenkoJoan OlivaVolha DzmitrukJoan Oñate NarcisoVolker SchirrmacherJoana A. LoureiroVong Binh LongJoana CaetanoVrinda GoteJoana F. B. BarataVuk UskokovićJoana FangueiroW. Jean DoddsJoana MagalhãesWael A. KhalilJoana PintoWael Hegazy‪Joana RoloWael MahdiJoana SimõesWael TraboulsiJoana ValenteWagner QuintilioJoan-Lluis Vives-CorronsWaldemar WagnerJoanna BartosińskaWalhan AlshaerJoanna BukowskaWalter TinganelliJoanna CzarneckaWanderley Pereira OliveiraJoanna DepciuchWangxiao HeJoanna FedorowiczWan-Jin ChenJoanna JaworskaWaquar AhsanJoanna KarbowniczekWaree TiyaboonchaiJoanna Lewandowska-ŁańcuckaWarren ViricelJoanna Skopinska-WisniewskaWasan KatipJoão Azevedo-SilvaWayne CarterJoão BassoWayne Thomas ShierJoão Carlos SilvaWei ChenJoão Henrique Ghilardi LagoWei ChengJoão Henrique LopesWei Guang SeetohJoão MartinsWei HuangJoão Ramalho-SantosWei ShaoJoão RodriguesWei WuJoaquim Esteves da SilvaWei XieJoe A. FralickWei-Chao LinJoe M. ViljoenWei-Dong XieJoeri L. AertsWeidong Zhou Johannes BoltzeWei-En YuanJohannes FruehWeifeng LinJohannes SchulzeWei-Hsiung YangJohn AdamovicsWeihua TianJohn B. MorrisWei-Hwa LeeJohn Cameron MillarWeijun QinJohn Dirk Vestergaard NielandWeilun TsaiJohn EisenbreyWeimer JörgJohn J. NemunaitisWeiwei HanJohn K. JacksonWeixiong LiangJohn MackWeiyun ShiJohn P. BannantineWen ChenJohn PowersWen ShiJohnny BullonWenbo ZhangJohnson M. LiuWen-Huei ChangJoias HammanWenjun ZhangJolanta B. ZawilskaWenjun WangJolanta RzymowskaWenliang SongJolanta TarasiukWen-Pin SuJomjai PeerapattanaWenqing ShuiJon Zárate SesmaWenquan YuJonathan L. CapeWensheng ZhangJonathan LovellWenting GuoJonathan Puente-RiveraWen-Tsong HsiehJong Bong LeeWenxin SongJong-Hwa JangWen-Yu PanJong-Suep BaekWerner WeitschiesJong-Wha JungWesley Lyeverton Correia RibeiroJoohee JungWessam H. Abd-elsalamJooho ParkWhocely Victor De CastroJoo-Young OheWided Najahi MissaouiJorddy CruzWilliam David TolbertJordi MartiWilliam Gustavo SganzerlaJordi OcañaWilliam J. TippingJörg BreitkreutzWilliam N. SetzerJorg DietrichWilliam RassmanJorge FeitoWilliam S. LawrenceJorge LopezWillibald WonischJorge SantosWilly M. J. M BogersJoris LammensWilman CarrilloJørn Bolstad ChristensenWing Shing HoJos L. EerselsWing-Hin LeeJos PaulusseWissem MnifJosé A. MartinsWitika BwalyaJosé A. S. CavaleiroWitold BrniakJose AndradeWitold JakubowskiJosé Antonio LupiáñezWładysław P. WęglarzJosé Antonio Morales-GonzálezWojciech JelskiJosé B. FariñaWojciech PiekoszewskiJosé Bernardo Noronha MatosWojnarovits LaszloJosé C. S. dos SantosWolfgang BinderJose CaaveiroWolf-Rainer AbrahamJosé Carlos AndradeWooram UmJosé CatitaWoosung KwonJose Escobar-ChavezWoubishet Zewdu TaffeseJosé Fernando Oñate-GarzónWouter L. J. HinrichsJosé L. Medina-FrancoWu YaoJose Luis Pérez-CastrillónWufu ZhuJosé Luis Rodríguez-FernándezWujie ZhangJosé Luis Viveros-CeballosWujun XuJosé María Palacios SantanderWuyi MingJosé Martínez LanaoXanthopoulos KonstantinosJose ParambilXavier BrazzolottoJosé Paulo Da SilvaXavier GasullJosé Pedraza ChaverriXavier MarsetJosé Pedro Cerón-CarrascoXavier Thankappan SuryabaiJosé R. A. FernandesXi ChenJosé Rafael De AlmeidaXi ZhaoJosé Ramón IsasiXianwen WangJose Ramon Moyano-MendezXiao XuJosé Ysmael Verde-GómezXiaodi ZhaoJosef FinstererXiaodong GeJoseph Calvin KouokamXiaodong ZhangJoseph CiccoliniXiaogang ZhangJoseph GovanXiaohua ZhuJoseph H. BanoubXiaohui ZhangJoseph LovecchioXiao-Kun OuyangJoseph PapamatheakisXiaoliang QiJoseph Robert MccorkleXiaolin ZiJosip HorvatXiaolong ChenJosipa BukićXiaonan FuJosipa VlainićXiao-Nan ZhangJosue SznitmanXiaoqiang WangJovana BradicXiaoqing MiaoJovanny GomezXiaowei DongJowita Samanta NiczyporykXiaowei ZengJozef Adam LiwoXiaoxing WangJozef Al-GousousXiaoyan ChenJózef DrabowiczXiaoyan WangJózef HaponiukXiaoyan Xu József SókiXiaoyu YanJuan Antonio Fafián LaboraXiaoyun TangJuan Aparicio-BlancoXiaxia DiJuan Bautista De SanctisXimeng SunJuan C. MejutoXiming GuoJuan CastilloXin ChenJuan David Ospina-VillaXin CuiJuan FraireXin HuangJuan GalloXin PanJuan Jose AcevedoXin SunJuan José Torrado DuránXin TongJuan LiXin ZhaoJuan Manuel Germán-AcacioXingang GuanJuan Pablo RigalliXinglei LiuJuan Pablo TosarXinyuan ChenJuan PellicoXiouras ChristosJuan SaavedraXiu-Juan ZhangJuan SantibanezXiupeng WangJudit OláhXuan MeiJudit VáradiXuan MuJudith González-ChristenXuenan GuJudith MihályXu-Fei WangJudyta Cielecka-PiontekXulin ChenJuergen ReichardtYahya SefidbakhtJui-Yang LaiYamin ZhuJulia LorenzoYamini KrishnanJulian Freen-van HeerenYan JinJulian QuodbachYan LeeJulie ConstanzoYan LiJulie FradetteYan Ming XuJulien RossignolYa-Na WuJulio A. Seijas VázquezYana ZorkinaJulio C. Rivera-LeyvaYanan WangJulio HenriquesYang LiJulita KulbackaYang MeiJulius BogomolovasYang TongJumpei SaitoYangcheng LuJun ChenYannis KaramanosJun FangYanyu HuangJun SawaiYaohua WeiJun ToyoharaYaoyao PengJunbo ZhangYaroslav O. MezhuevJun-Bom ParkYasuhiko TabataJunchao TongYasuhiro KosugeJung Hwan ParkYasuhiro TsumeJung Seung LeeYasuko ObataJung Su RyuYasumasa OkazakiJung-Eun ParkYasushi AranoJung-Jae LeeYau Kei ChanJun-Goo JeeYavuz Nuri ErtasJung-Woo BaeYeimmy Peralta-RuizJung-Woo ChaeYeliz DemirJunjie LiYeon Sun LeeJunmei ZhangYi LuJunseong ParkYi ZuoJunzi WuYibin ZhangJuraj PiestanskyYibo QiuJu-Seop KangYi-Cheng ChenJustin S. AntonyYifei JinJustyna Gornowicz-PorowskaYifeng CaoJya-Wei ChengYihenew Simegniew BirhanJyothsna ManikkathYi-Hsien HsiehK. G. PrasanthYimei JiaKa L. HongYing Bin YanKa YangYing LiuKai XiaoYing QinKai ZhangYing YangKai ZhaoYinghuai ZhuKai-Jun LuoYining XuKailash SinghYitang SunKajal GhosalYi-Ting ChiangKalas WojciechYiwei TianKalyan RameshYiwen LiKalyani NairYiwen ZhaoKamal Hany HusseinYlenia ZambitoKameliya AnichinaYoann CazaubonKamil GareevYogeshvar N. KaliaKamil KaminskiYohann CorvisKamil PiskaYohei KotsuchibashiKamila MyszkaYohei SekinoKankan WangYoichi ShimizuKaran AroraYolanda SalinasKarel AllegaertYolanda Terán-FigueroaKarem A. CourtYolanda VidaKaren Ildico Hirsch-ErnstYong LiuKaren KhachatryanYong TangKaridia KonateYong-Ai XiongKarla Juarez-MorenoYongchel AhnKarmen StankovYongfen XuKarnaker TupallyYongfeng ZhaoKarol KyziołYong-Jiang LiKarol P. NartowskiYong-Moon LeeKarol SteckiewiczYongping BaoKarol WróblewskiYongqiang LiuKarola Vorauer-UhlYongtai ZhangKatalin KristóYongwoo JangKatarina SmilkovYoshi OdakaKatarína ValachováYoshiaki SunamiKatarzyna Bialik-WąsYoshihiro ShidojiKatarzyna D SluzalskaYoshihisa HashiguchiKatarzyna GobisYoshikane YamauchiKatarzyna Grzelak-BłaszczykYoshikatsu KogaKatarzyna JelonekYoshikazu TokuokaKatarzyna Kosicka-NoworzyńYoshiki KatayamaKatarzyna Małolepsza-JarmołowskaYoshio TakesueKatarzyna MichalskaYoshito IkadaKatarzyna RoszekYosif AlmoshariKatarzyna TureckaYosuke HashimotoKatarzyna WigluszYotsanan WeerapolKatarzyna ZawadaYoung G. ShinKatharigatta Narayanaswamy VenugopalaYoung Ho KimKatharine CarterYoung Whan ChoiKathleen HefferonYoung-IL JeongKathleen PishasYoung-Ji ShiaoKathrin SpendierYousuf MohammedKathryn Y. BurgeYu ChenKatia SeremetaYu Chul KimKatie L. PenningtonYu FengKatona GáborYu ZhangKatshito NagaiYuan TangKatsuhiko ArigaYuanzhe LiKatyayani TatipartiYub Raj NeupaneKaushalendra ChaturvediYubin ZhouKavindra K. KesariYu-Chan ChangKay-Dietrich WagnerYu-Chieh ChenKazem NazariYu-Chih ChenKazım KöseYu-Chih ChiangKazuaki YoshimuneYu-De ChuKazuhisa MatsunagaYue GuiKazuhito NakaYue SuKazuki MatsuiYue WangKazutaka HigakiYuhao LiKazutaka HirakawaYuhao QiangKazuya MaedaYuji IsegawaKei MaruyamaYuji UchioKei NishidaYuji WangKeiji ItakaYujie SuKeitaro SouYukihiro HamadaKeith R. FoxYulia PushkarKeith RochfortYu-Ling LinKelong FanYuliya RyzhkovaKen LeungYun LiuKendal RyterYun ShiKeng-Shiang HuangYun-Bae KimKenichi GotoYunchen YangKen-Ichiro MatsumotoYunfeng ZhaoKening LangYung-Kang ShenKenneth W. McMillinYunhai CuiKenry KenryYunhua GaoKenta MoritaYu-Ning TengKeqiang ZhangYuning ZhangKerstin MenckYunjin JungKeshav Raj PaudelYunlu DaiKeshen LiYunqian DaiKetan M. RanchYunus Emre BeyhanKetan PatelYun-Wen ZhengKeumhan NohYuriy L. OrlovKeun Sik KimYu-Ru ShihKevin BattyYury TorubaevKevin ItaYusuf HaggagKevin LiYusuke SatoKevin WardYuta YoshizakiKeyvin DarneyYutaka InoueKhac-Minh ThaiYutaka MatsudaKhair AlharethYutaka OhsedoKhaled A. El-TarabilyYutian MaKhaled A. ShaabanYu-Tse WuKhaled ElokelyYuvaraj ArunKhaled HosnyYuyan JiangKhaled M. DarwishYves-Jacques SchneiderKhalid HaddiZacharoula IatridiKhalid KarrouchiZachary WarnkenKhalid Mohamed El-SayZahari VinarovKhalid RazaZahra LotfollahiKheireddine El-BoubbouZahra RattrayKhrystyna HarhayZaida OrtegaKhurshid AhmadZain-ul AbadinKhushwant Singh BhullarZaiqun YuKi Cheong ParkZakia A. OlamaKi Hyun NamZar Chi SoeKi Su KimŽarko MitićKibeom KimZbigniew RaszewskiKieu The Loan TrinhZbigniew RybakKim Lambertsen LarsenZbigniew StojekKim Van VlietZdenek KejikKimberly PerezZdenka PeršinKimberly ShepardZdeňka ŠklubalováKiran SandhuZebunnissa RamtoolaKiran TotiZeenat IqbalKiriaki PavlidouZeenat MirzaKirill V. ZaitsevZeeshan HamidKirsten J. M. SchimmelZehra EdisKiruphagaran ThangarajuZehuan HuangKishor WasanZeid A. NimaKit Leong CheongZeljka VanicKitherian SahayarajŽeljko PetrovskiKitiporn PlaimasZeljko SundovKlaus MerzZengdi ZhangKlemen BohincZengying QiaoKleoniki GiannousiZening WangKlepetsanis PavlosZhanna NazarkinaKoen VermeulenZhao WangKoichi IgarashiZhao YangKoichi KitagawaZhaohua DaiKoji MatsuokaZhaojun WeiKoki KamiyaZhaowei ChenKollur Shiva PrasadZhaoxi SunKondareddy CherukulaZhaozhu ZhengKonrad KowalskiZhe LiuKonstantin A. LukyanovZhe YangKonstantin VolchoZhebao WuKonstantinos DimasZhengguo Wu Konstantinos GardikisZheng-Rong LuKonstantinos PapadimitriouZhengwei HuangKonstantinos StamatopoulosZhengzhong XuKormos ViktóriaZhi ShiKornél KirályZhichao LouKorzhikova-Vlakh EvgeniaZhicheng YaoKoyo NishidaZhidong ZhouKrish KrishnanZhifeng DuKrishna ChaitanyaZhifeng JingKrishna K. SharmaZhijie ChangKrishna Mohan SurapaneniZhiping FengKrishna PatelZhiping XuKrishna YadavZhiqiang YuKrishnendu PalZhiyong ChenKristin KöppenZhiyong HeKristina NesporovaZhongwei LiuKristina PavicZhu HuiKristina RadoševićZhuoran MaKristina RamanauskienėZhuqiu JinKristóf Endre KárolyZhuxian ZhouKrisztina Takács-NovákZia ArifKrystyna Mojsiewicz-PieńkowskaZili GuKrzesimir CiuraZili SideratouKrzysztof BilminZinaida B. ShifrinaKrzysztof GorynskiZiwen JiangKrzysztof OwsianikZiwen WangKrzysztof SztankeZiyad A. Alrowaili Krzysztof TomczukZiyue LiKrzysztof WalczyńskiZlatko KopeckiKsenia EgorovaZofia MazerskaKsenia V. DrozdZofia Urbanczyk-LipkowskaKuang-Ming KuoZoltán BánócziKuan-Han LeeZoltan KovacsKuldeep GuptaZoltán ÚjhelyiKuldeep K. BansalZoltan VargaKuldeep Singh AttriZonghai ShengKumar GanesanZongjie WangKummara Madhusudana RaoZongxiang TangKun LiangZoran MinicKun ZhouZoran ZekovićKunal PalZorana MilanovicKun-Eek KilZoubir DjeradaKunlei WangZrinka RajićKuo-Ching MeiZsófia Edit PápayKuo-Shyan LinZsolt BacsoKuthati YaswanthZsuzsanna GáborikKwang Suk LimZuhai LeiKyeong-Ryoon LeeZuoxu XieKyoung Sub KimZu-Quan HuKyriaki KyriakidouZuzana BenkováKyriakos KachrimanisZvezdelina Lyubenova Yaneva


